# Sacroiliac joint fusion guided by intraoperatively superimposed virtual surgical planning using simulated fluoroscopic images

**DOI:** 10.1016/j.bas.2024.102905

**Published:** 2024-08-02

**Authors:** Steven Lankheet, Nick Kampkuiper, Jorm Nellensteijn, Edsko Hekman, Gabriëlle Tuijthof, Femke Schröder, Maaike Koenrades

**Affiliations:** a Medical 3D Lab, Medical Spectrum Twente, Enschede, the Netherlands; b Department of Orthopedic Surgery, Medical Spectrum Twente, Enschede, the Netherlands; c Department of Biomechanical Engineering, Faculty of Engineering Technology, Technical Medical Center, University of Twente, Enschede, the Netherlands; d Multi-Modality Medical Imaging (M3i) Group, Faculty of Science and Technology, Technical Medical Center, University of Twente, Enschede, the Netherlands

**Keywords:** Simulated fluoroscopic images, Virtual surgical planning, Computer-assisted surgery, Minimally invasive sacroiliac joint fusion

## Abstract

**Introduction:**

Sacroiliac joint fusion (SIJF) is a minimally invasive treatment for sacroiliac (SI) dysfunction. It involves placing implants through the SI joint under fluoroscopic guidance, requiring precise implant positioning to avoid nerve injury. Preoperative virtual surgical planning (VSP) aids in optimal positioning, but replicating it accurately in the operating room is challenging.

**Research question:**

This study aims to assess the feasibility of superimposing VSP onto intraoperative fluoroscopic images to aid in optimal implant placement.

**Material and methods:**

A method for intraoperative guidance using 3D/2D registration was developed and tested during SIJF as an available and potentially efficient alternative for costly and more invasive navigation systems. Preoperatively, a VSP is performed and simulated fluoroscopic images are generated from a preoperative CT scan. During surgery, the simulated image that visually best matches the intraoperative fluoroscopic image is selected. Subsequently, the VSP is superimposed onto the intraoperative fluoroscopic image using a developed script-based workflow. The surgeon then places the implants accordingly. Postoperative implant placement accuracy was evaluated.

**Results:**

Five interventions were performed on five patients, resulting in a total of 15 placed implants. Minor complications without clinical consequences occurred in one case, primarily attributable to the patient's anatomy and pathological manifestations. Mean deviations at implant apex and 3D angle were 4.7 ± 1.6 mm and 3.5 ± 1.3°, respectively.

**Discussion and conclusions:**

The developed intraoperative workflow was feasible and resulted in implants placed with low deviations from the VSP. Further research is needed to automate and validate this method in a larger cohort.

## Highlights

• 2D fluoroscopic guidance is commonly used in sacroiliac joint fusion, but it presents challenges for correct implant placement.
• Superimposition of a virtual surgical planning greatly enhances intraoperative guidance.
• The new method may benefit the procedure by providing additional guidance during surgery.
• By superimposing virtual surgical planning, implant placement may be more accurate, reducing the risk of complications.
• The workflow was found to be feasible for clinical use and resulted in acceptably low implant placement deviations.

## Graphical abstract

**Figure:**
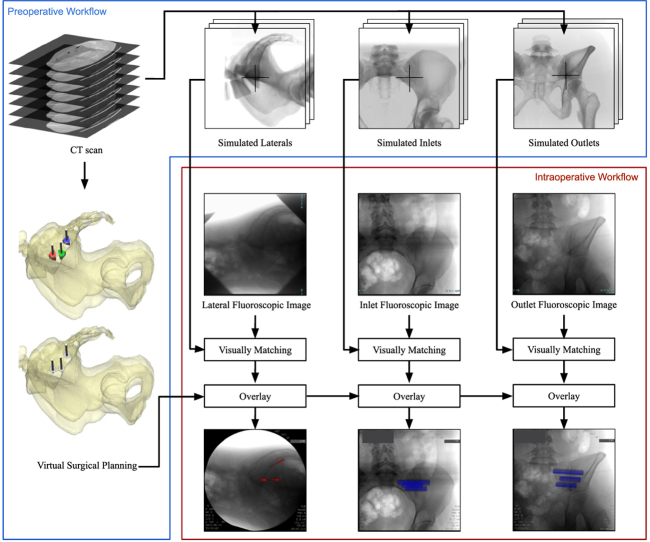

## 1 Introduction

Sacroiliac (SI) joint dysfunction can cause debilitating low back pain, which may be caused by pregnancy, trauma, hyper- or hypomobility syndromes or previous lumbar fusion surgery. Sacroiliac joint fusion (SIJF) can be performed to immobilize the SI joint and thereby eliminate motion, which plays an essential role in the nociception mechanism in patients suffering from SI joint dysfunction (Rashbaum et al., 2016).

This paper focuses on minimally invasive SIJF through a lateral approach using cannulated triangular titanium implants. Guide pins for positioning the implants are generally placed freehandedly through the SI joint under 2D fluoroscopic guidance. Due to this 2D representation of the volume, the surgeon frequently switches between lateral, inlet, and outlet views to confirm safe positioning of the guide pins. While placing the guide pins, damage to critical structures needs to be avoided. Breaching of the sacral ala may affect the L4, L5, and obturator nerves, and protrusion into the S1 and S2 foramen may affect the S1 and S2 nerves (Grechenig et al., 2021). Damage to these critical structures can result in major complications, such as nerve impingement or nerve damage, resulting in radiating pain, numbness, or palsy (Berger-Groch et al., 2020; Schoell et al., 2016). Malpositioning of implants may also lead to an insufficient bone-implant contact surface, which may affect the ingrowth of the implants and the stability of the fusion. Furthermore, configuration of the implants is a key component for the stability of the fusion. Placement of the implants the most stable configuration results in less range of motion of the SI joint, possibly resulting fewer reinterventions due to increased stability and therefore lower chance of implant loosening (Freeman et al., 2021; Lindsey et al., 2018). Unfortunately, due to the lack of 3D spatial information, it can be challenging to place the implants in the optimal and safe position (Schippers et al., 2021).

Placing the implants without damaging the critical structures is challenging due to the varying shape of the foramen, the SI joint and the sacrum (Cheng and Song, 2003; Jesse et al., 2017; Mahato, 2016). Therefore, we expect that additional intraoperative guidance could reduce the risk of complications.

Navigation guided techniques may decrease the risk of complications and allow for better implant placement (Polly and Holton, 2020; Naik et al., 2022; Piche et al., 2021). However, these systems are not commonly used for SIJF, costly, more invasive, and may unnecessarily increase the duration of the surgery (Naik et al., 2022). As an alternative, we developed a method for virtual surgical planning (VSP) in SIJF in a previous study, which requires no additional hardware in the operating room (Kampkuiper et al., 2023). This previously developed approach enables the visualization of a patient's anatomy in 3D, facilitates preoperative planning for the most optimal patient-specific implant configuration, and intraoperatively utilizes simulated fluoroscopic images to guide implant placement.

However, in this previously developed approach, the visualization of the planning was created preoperatively in a predetermined view, presenting a notable challenge in achieving intraoperative fluoroscopic images that correspond to the predetermined view. Because the 3D planning can be visualized in a different view, it still may be challenging to reproduce the planned implant positions accurately.

To improve the previous developed VSP, one potential solution involves projecting the VSP onto the intraoperative fluoroscopic image. This can be achieved by preoperatively generating multiple simulated fluoroscopic images and selecting the one that best matches the intraoperative image in the OR. The best matching simulated fluoroscopic image can be registered to the intraoperative fluoroscopic image (Markelj et al., 2012; Saadat et al., 2022). Recent research shows that this simulated fluoroscopy method can be applied for SIJF to highlight critical structures such as the foramina (Schippers et al., 2021). This study aims to implement this simulated fluoroscopy method in clinical practice and demonstrate the feasibility of using simulated fluoroscopic images to register VSP on intraoperative fluoroscopic images to assist in optimal implant placement as an alternative for navigation guided systems.

## 2 Methods

The workflow to assist implant placement using VSP and fluoroscopic 3D/2D registration was developed and implemented in a clinical setting. To evaluate the new workflow, the clinical outcome, implant placement accuracy, clinical feasibility, and expert opinion were taken into account. In November 2021, the workflow was applied in one proof of principle case. After this successful case, four more patients were prospectively included in June 2023. All five patients were diagnosed with SI joint dysfunction and scheduled for SIJF (all women and 60% of the surgeries were left-sided). One surgeon (JN), with approximately 130 prior SIJF surgeries using conventional 2D fluoroscopic guidance, performed all procedures. The third case had abnormal anatomy, including a pubic bone resection fourteen years ago and a malformed S1 body on the operated right SI joint. However, the case was not excluded since different sacral morphologies are common. Ethical approval for all clinical evaluation studies where 3D technology was used was obtained in July 2022.

Furthermore, superimposing the VSP onto intraoperative imaging was considered to have no additional risk compared to the use of VSP alone. Written informed consent was waived for patients included before this date and those included hereafter gave written informed consent. As standard clinical care, a preoperative CT scan (slice thickness 1 mm; pixel spacing 0.74–0.84 mm, tube voltage 100–120 kV) of the pelvis was acquired using a dual-source Somatom Definition Flash CT scanner (Siemens Healthcare, Erlangen, Germany).

### 2.1 Virtual surgical planning

Based on preoperative CT scans, VSPs were created according to the previously published work by Kampkuiper et al. (2023). VSP for SIJF is standard clinical care in Medisch Spectrum Twente (MST, Enschede, The Netherlands) and it is made using the medically certified software environment of Materialise Mimics 25.0 (Materialise, Leuven, Belgium). First, the pelvis was segmented and subsequently a 3D model of the pelvis was created. Then, the three triangular implants were virtually inserted into the CT volume, through the SI joint, oriented perpendicular to a true lateral view of the 3D model of the pelvis. Based on the CT data the implants were placed in a parallel triangular patient-specific implant configuration while generally maintaining at least a safety margin of 3 mm between the implants and the cortex of the sacrum (anterior cortex and cortex around the neural foramina).

### 2.2 Technical description of the workflow

Our method involves presenting the VSP to the surgeon using copies of the actual intraoperative fluoroscopic images of lateral, inlet and outlet view. Rather than painstakingly adjusting the C-arm until exact lateral, inlet and outlet views are obtained, we accept the practically obtainable fluoroscopic images. Then, the best matching simulated fluoroscopic image is obtained from a large number of prepared simulated images taken from slightly different directions. To superimpose the VSP—i.e., the implants, guide pins, and/or entry points—onto the intraoperative image, a simulated fluoroscopic image of the planning is created according to the orientation of the best matching fluoroscopic image.

**Fig. 1:**
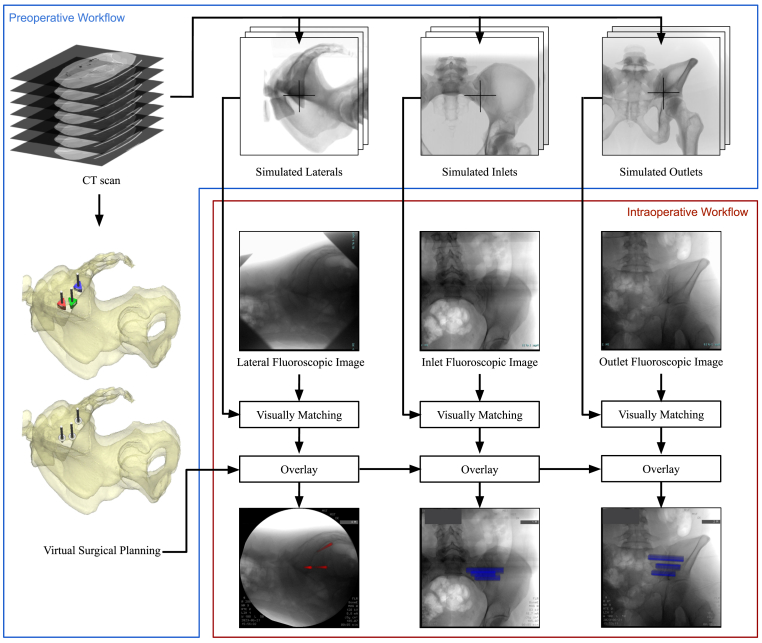
A schematic overview of the proposed workflow for superimposing the virtual surgical planning (VSP) during surgery, comprising a preoperative and intraoperative part. Preoperatively, VSP and series of simulated fluoroscopic images are generated. The central ray of the X-ray beam (center of rotation) is depicted with crosses in the simulated fluoroscopic images. The intraoperative part shows the matching of the fluoroscopic images with a simulated fluoroscopic image and superimposing the guide pins (red) and implants (blue) on the intraoperative fluoroscopic image. (For interpretation of the references to color in this figure legend, the reader is referred to the Web version of this article.)

The workflow that superimposed the VSP onto the intraoperative fluoroscopic imaging was also developed within Materialise Mimics 25.0. The additional Scripting module of Materialise Mimics (using Python 3.8) enables the creation of supplementary functions and a graphical user interface (GUI) (Python Software Foundation). An overview of the preoperative and intra-operative workflow is shown in Fig. 1.

The workflow consists of six steps. Step 1, the developed scripted workflow guides the user preoperatively in finding the starting orientation for the lateral, inlet and outlet views by rotating the 3D model of the pelvis. Step 2, these starting orientations are used to preoperatively create simulated fluoroscopic images using the fluoroscopy module of Materialise Mimics. The simulated images must be generated with a standardized center of rotation, ensuring a fixed distance between the patient and the X-ray source, as well as a fixed position for the central ray. Selecting the exact same center of rotation intraoperatively is necessary to make comparable simulated fluoroscopic images due to the divergence of the X-ray geometry. For the lateral view, the selection of this center of rotation is in the sacral S1 segment at an intersection point with the iliac cortical densities, i.e., landmarks that are visible on a lateral X-ray of the pelvis. In the case of the inlet and outlet view, this center is located at the height of the SI joint gap. These centers of rotation are depicted in the simulated fluoroscopic images in Fig. 1. Step 3, the software automatically creates a range of simulated fluoroscopic images within specified intervals with the 3D models, starting orientation, and center of rotation as input (preoperative script, Supplementary files I). Step 4, the intraoperative true fluoroscopic images acquired during the interventions, must be imported into the software. Step 5, the user selects the visually best-matching simulated fluoroscopic image for the lateral, inlet, and outlet views using a created GUI (intraoperative script, Supplementary files II). The orientation of the best-matching simulated fluoroscopic image is used to create a simulated fluoroscopic images of solely the guide pins, entry points, and implants. These elements are positioned in the same coordinate system as the CT data and 3D model of the pelvis, that are used to create the simulated fluoroscopic images. Step 6, to register, i.e., superimpose, the VSP to the intraoperative fluoroscopic image, a rigid point set registration method was used (Python Software Foundation). This involves manually selecting well-defined landmarks in the intraoperative fluoroscopic image and simulated fluoroscopic image to subsequently overlay these images automatically. The simulated guide pins (red), entry points (blue), or implants (blue) are depicted in color to enhance contrast in the resulting image. The views and selection of superimposed objects can be switched intraoperatively. A supplementary video is available online (Supplementary materials III), demonstrating the intraoperative use of the script-based workflow.

### 2.3 Intraoperative clinical workflow

**Fig. 2:**
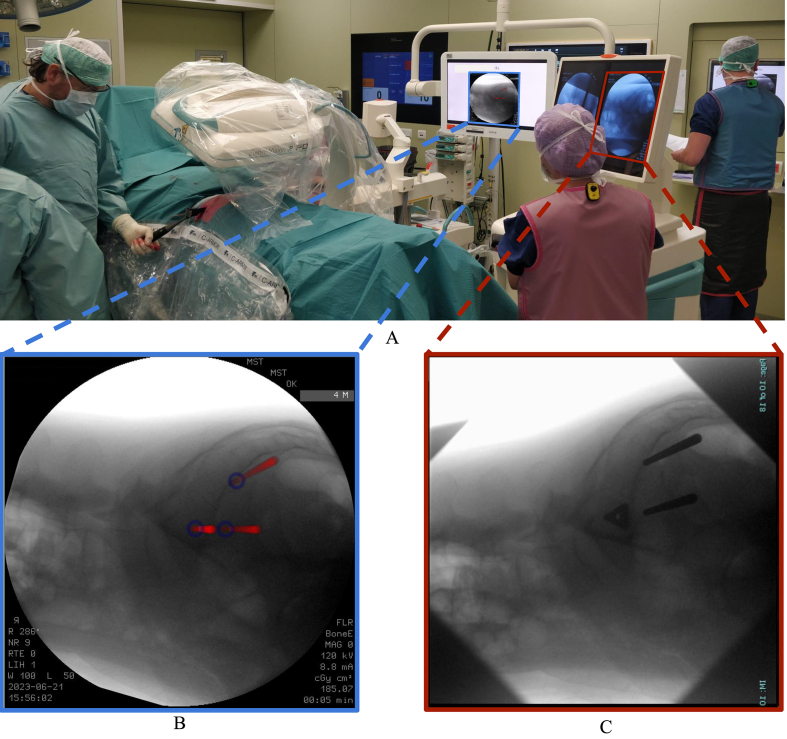
Overview of the operating room (A), where the left screen displays the intraoperative fluoroscopic image with superimposed guide pins (red) and entry points (blue) from the virtual surgical planning (B) and right screen is the monitor of the fluoroscopy device after the first implant was placed (C). (For interpretation of the references to color in this figure legend, the reader is referred to the Web version of this article.)

At the start of the surgical procedures, the patients were positioned in a prone position under general anesthesia. A vacuum mattress was used to ensure the patient's stable positioning. Next, the C-arm was positioned to acquire a lateral fluoroscopic image of the pelvis using a Ziehm Vision FD C-arm (Ziehm Imaging GmbH, Nuremberg, Germany). To make the intraoperative fluoroscopic images comparable to the simulated fluoroscopic images that were preoperatively generated, the center of rotation was intraoperatively aligned with the previously determined position on the sacrum (as in the simulated fluoroscopic images in Fig. 1). After the lateral view, inlet and outlet fluoroscopic images were obtained. These images were loaded into the script by transferring the data using a USB drive along with the prepared simulated images. The visually best-matching simulated fluoroscopic image was manually selected for each view. This was achieved by critically comparing several anatomical landmarks in both images. For the lateral view, landmarks such as iliac cortical densities, iliac crests, and greater sciatic notches were used. These landmarks appear differently in all images due to the angulation of the simulated X-ray beam and the divergence of X-ray geometry. In the inlet and outlet views, respectively, similarity in the anterior sacral walls and the position of pubic bone relative to the sacral foramen are crucial. After the best matching images were found, manually selected landmarks were used for the image-to-image registration to create an overlay of the planned entry points, guide pins, and implants onto the intraoperative fluoroscopic images. The created overlay was then depicted on a screen next to the monitor of the C-arm during surgery (Fig. 2). The surgeon is able to switch between the lateral, inlet and outlet view and can also select what objects of the VSP should be shown, i.e., guide pins, entry points or implants. The surgery was then performed as usual, with the addition of using the fluoroscopic image with the overlaid VSP as a reference for the positioning of the guide pins and implants. The guide pins were first positioned at a safe depth, whereafter the surgeon assured safe and correct placement by fluoroscopic guidance before proceeding with the positioning of the triangular implants (iFuse Implant System, SI-Bone, Santa Clara, USA) over the guide pins (Fig. 2C). Finally, the guide pins were retracted and the incision was closed.

### 2.4 Performance evaluation

To evaluate the performance of the workflow several outcomes were assessed. First, routine postoperative CT scans were used to determine whether the implants were safely placed without malpositioning complications. These postoperative CT scans were made with the same scanner and parameters as the preoperative scan, except that the tube voltage was increased to 140 kV to reduce metal induced image artifacts. Second, the implant placement accuracy was evaluated by determining the positions of the implants in routine postoperative CT imaging and comparing these to their position in the VSP. The method to determine the implant placement accuracy is elaborated in Kampkuiper et al. (2023). Third, to assess the clinical feasibility the intraoperative processing times, registration errors, and resulting images were examined. The intraoperative processing times were assessed to determine whether the new workflow influenced the procedure time. If the intraoperative process can be performed while the sterile field is created and the incision is made, it is assumed that the workflow does not delay the procedure.

Registration errors of the rigid point set registration method were evaluated by analyzing mean error and standard deviation. Postoperatively, the intraoperative fluoroscopic images, intraoperative fluoroscopic images with overlaid VSP, intraoperative fluoroscopic images with actually inserted guide pins or implants, and the VSP matched with postoperative 3D models were examined for similarity to gain an impression of the feasibility. Additionally, implant placement accuracy was visually assessed in the preoperative 3D model of the pelvis. Finally, to gain an impression of the added value from the surgeon's perspective, the surgeon (JN, 130 prior SIJF surgeries) was asked to provide an expert opinion on the following questions: “Did the developed workflow provide additional guidance during surgery?”, “What is are the benefits of superimposing VSP onto intraoperative fluoroscopic imaging?”, “Does the workflow improve the efficiency of the procedure?”, and “Did you experience any difficulties in the new workflow?".

## 3 Results

The developed 3D/2D registration workflow was successfully used in a clinical setting during five SIJF surgeries. In one case, creation of the VSP revealed that narrow anatomy did not allow parallel placement of three implants. To be able to place three implants, it was chosen to place the first implant under a posterior inclination.

### 3.1 Clinical outcomes

As evidenced by postoperative CT imaging, two minor complications occurred in one case, i.e., the case with abnormal anatomy. The first implant, i.e., the one located most cranially, slightly breached the anterior wall of the sacrum and an iliac fracture occurred between the first and second implant. Besides a possible increase in postoperative pain, neither of these complications required surgical reintervention or had longterm clinical consequences. No complications occurred in the other cases. All patients experienced a reduction in pain complaints after the intervention.

### 3.2 Implant placement accuracy

**Fig. 3:**
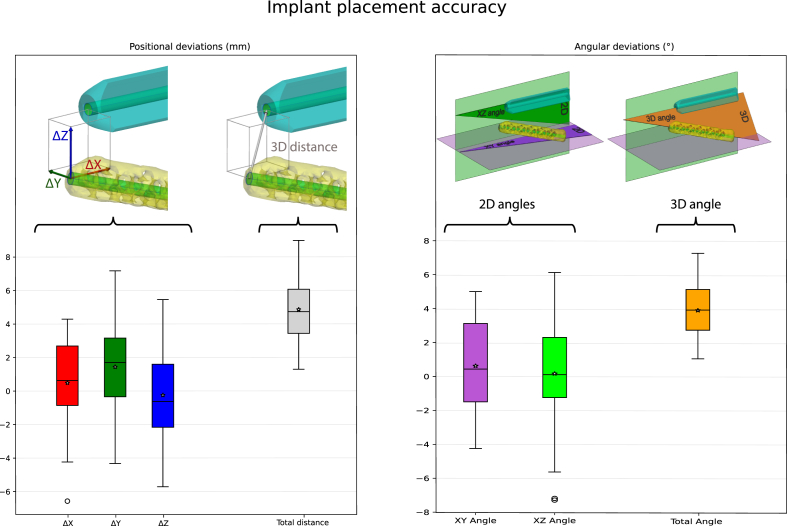
The implant placement accuracy measures are visually displayed in the boxplot. In the top images, the preoperatively planned implant is depicted in cyan and the postoperatively placed implants are depicted in yellow. In these images, the magnitude of the deviation is intentionally amplified in order to enhance visual clarity. The boxplots containing the seven implant placement accuracy measures of fifteen implants (five cases). The colors of the boxplot match to the colors of the deviation the top images. In the left boxplot of the positional deviations are shown and in the right boxplot the angular deviations. The boxes represent the median, quartile 1 and quartile 3. Whiskers extend from the box to the most extreme data points within 1.5 times the inter quartile range, and individual points beyond this range are considered outliers and are plotted separately. Additionally, the mean values are depicted using asterisks. (For interpretation of the references to color in this figure legend, the reader is referred to the Web version of this article.)

A total of fifteen implants were inserted, three implants per patient. The placement accuracy of all fifteen implants is shown in Fig. 3. The mean 3D distance deviation at the apexes of the implants was 4.7 ± 1.6 mm and the mean 3D angular deviation was 3.5 ± 1.3°.

### 3.3 Clinical feasibility of the workflow

**Fig. 4:**
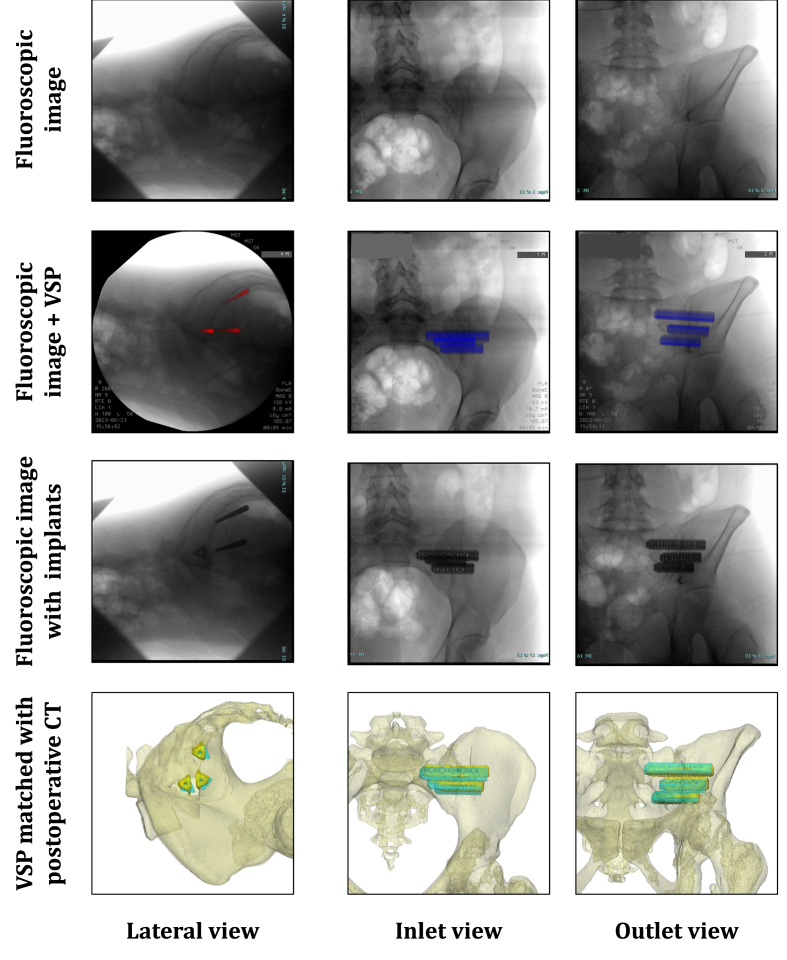
Overview of visualization of fluoroscopic images, fluoroscopic images with superimposed virtual surgical plan, fluoroscopic images with inserted guide pins or implants, and 3D models with planned an actually placed implants of one case. The postoperative evaluation in the bottom row shows the surgical plan, i.e., pelvis and planed implant positions (cyan) and the postoperative implant positioning (yellow). The bones are displayed transparently to enhance the visibility of the implants. (For interpretation of the references to color in this figure legend, the reader is referred to the Web version of this article.)

An example of the simulated fluoroscopic images, intraoperative fluoroscopic images, and 3D models of one case are shown in Fig. 4. Notably, in all cases the resulting image was not a true lateral view because the patient or C-arm had been slightly tilted. However, in these images, the iliac cortical densities were properly aligned with respect to each other. During surgery, it took approximately 5 min to export the three fluoroscopic views, find the visually best-matching simulated fluoroscopic images, and present the intraoperative fluoroscopic image with superimposed VSP on the screens in the operating room (Fig. 2A). Manual rigid point set registration of the simulated fluoroscopic image with the intraoperative fluoroscopic image showed a mean error of 1.4 ± 1.0 mm. The resulting images with superimposed VSP showed visually adequate correspondence to the simulated fluoroscopic images. Furthermore, the preoperative 3D models and actually inserted implants also showed correspondence.

### 3.4 Expert opinion

In the expert's (JN) opinion, the VSP superimposed onto intraoperative fluoroscopic images can provide additional guidance and simplify the procedure. It was observed that the workflow improved the efficiency of the procedure compared to the previous method with preoperatively prepared simulated fluoroscopic images without intraoperative registration (Kampkuiper et al., 2023). The current workflow adjusts the VSP to the intraoperative image instead of recreating the exact same C-arm orientation as described in the previous method (Kampkuiper et al., 2023). Furthermore, the surgeon indicated that the workflow, as presented in this study using multiple simulated fluoroscopic images, allows increased efficiency in the placement of the guide pins. The surgeon experienced that, in some cases, it was observed that either the fluoroscopy device or the patient was slightly tilted during the creation of the sterile field or during the procedure. This caused a slight discrepancy between the intraoperative fluoroscopy image that was used for the overlay and newly made intraoperative fluoroscopic images. In case this occurred, the surgeon tried to adjust the set-up in order to mimic the fluoroscopic image that was used to create the overlay.

## 4 Discussion

This study demonstrated the feasibility of a workflow that superimposed VSP on intraoperative fluoroscopic images using simulated fluoroscopic images to guide implant placement as an alternative for navigation guided systems. This alternative can be faster, does not require additional hardware beyond a C-arm, and is less invasive since no reference markers are needed. Results show that the workflow allowed the placement of implants without major malpositioning complications, even in the case of challenging anatomy. The implants were placed with relatively high accuracy and within the regular operation time. The developed workflow was created using CE-certified medical software and can be used in clinical practice. It guides the surgeon during the reconstruction of the VSP.

**Fig. 5:**
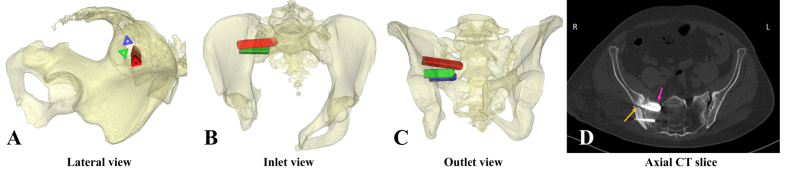
A 3D visualization of the Virtual Surgical Planning (VSP) of the third case in lateral, inlet and outlet view (A–C). It can be seen that the first (red) is placed with a posterior inclination and that the second (green) and third (blue) are placed perpendicular to the true lateral view. The uncommon sacral morphology, hemi-sacralization left and malformed S1 body right, as well as the severe arthritis of both SI joints can be seen. In the axial CT slice (D), a minor breach of the anterior sacrum (highlighted by the orange arrow) and an ilium fracture (pink arrow) are depicted. (For interpretation of the references to color in this figure legend, the reader is referred to the Web version of this article.)

In four out of five cases, no complications occurred. In the other case, two minor complications occurred (Fig. 5. D), which are most likely to be attributable to pre-existing anatomical and pathological manifestations. The anatomical variation led us to place the first implant under a slightly posterior inclination (Fig. 5 A-C). Usually, when solely 2D fluoroscopic guidance is used, placing the implants under an angle is not preferred since this is exceedingly challenging. However, this was done in this case to ensure optimal implant-bone contact and accommodate three implants, thus avoiding potentially inferior results associated with only two implants (Freeman et al., 2021; Lindsey et al., 2018). As mentioned, this approach would have been more risky using only 2D fluoroscopic guidance. Therefore, the developed workflow was exceptionally useful in this complex case, as it provided additional guidance in placing the implant with the planned inclination.

The current study demonstrated a mean positional deviation of 4.7 ± 1.6 mm and a mean angular deviation of 3.5 ± 1.3°. Considering it is a freehanded approach we think that this is a relatively high accuracy. Furthermore, not only the magnitude but also the direction of the deviation is of importance. The found deviation is not necessarily in the direction of a critical structure and may point in a safe direction, e.g. to a lesser depth, as the surgeon can adjust the placement according to subsequent intraoperative images. Apart from our previous study we found no other literature concerning the implant placement accuracy of SI implants. In our previous study with implementation of VSP in ten cases, the overall mean implant placement accuracy was 4.9 ± 1.26 mm and 4.0 ± 1.44° (Kampkuiper et al., 2023). In this method, a 2D visualization (e.g. a simulated fluoroscopic image) of the 3D planning was created preoperatively in a predetermined view. The method in the current article improves upon our previous work by visualizing the 3D planning in the same view as the intraoperative fluoroscopic image. The observed difference in implant accuracy may indicate that the current workflow slightly increases accuracy. However, due to the limited number of patients, no statistical analysis can be performed to confirm this observation. The mean positional deviation for the first (proof of principle) and third (abnormal anatomy) cases were 4.2 mm and 5.4 mm, respectively. For these two cases, the mean angular deviations were 3.9° and 4.2°, respectively. These two cases do not seem to deviate from the other three cases.

The newly developed workflow did not delay the surgical procedure. The processing time, from exporting the fluoroscopic images to displaying the resulting images on a monitor, did not affect the timeline, as it aligns with the approximately 5 min required for creating the sterile field and making the incision. However, an additional person is required to perform the processing steps. Due to the limited number of included patients and influence of procedure time related confounders (BMI and anatomy), no comparisons were made to the procedure time of conventional treatment or navigation-guided treatment. This study's manual registration of the simulated fluoroscopic image with the intraoperative fluoroscopic image showed a mean error of 1.4 ± 1.0 mm. Compared to Schippers et al.'s study, a smaller registration error was found (lateral 5.2 ± 2.7 mm, inlet 2.7 ± 2.0 mm, and outlet 1.9 ± 0.8 mm) (Schippers et al., 2021). This difference is probably due to their exclusion of the X-ray magnification, as they employed a line source instead of a point source. The current workflow can create simulated images that are visually comparable to the intraoperative fluoroscopic images (Fig. 4.).

During surgery, it was noted that in some cases, the best matching view was not a true lateral view, despite alignment of the iliac cortical densities. This underscores the difficulty of intraoperatively creating a true lateral view. In certain patients, the iliac cortical densities may be less pronounced or not pronounced at all. This further highlights the importance and necessity of superimposing the VSP onto obtained intraoperative images and not on predetermined views.

The expert mentioned that the workflow provided additional guidance and seemed to increase efficiency. A mentioned drawback was that the patient or the C-arm could accidently be moved during the procedure, causing a discrepancy between the created overlay and the subsequent fluoroscopic images.

A limitation of the workflow was the inability to quantify the error in selecting the visually best matching simulated fluoroscopic image. This, along with the other manual registration errors, can be eliminated by further automating the workflow. Automated matching and registration methods could superimpose the VSP over each image directly, thus visualizing the VSP in real-time. Implementing automatic registration could decrease registration errors, enhance workflow efficiency, and increase implant placement accuracy. Currently, the manual software workflow requires a trained user. One way to automate the process is to use Deep Learning to obtain the angle and distance from a created fluoroscopic image (Kausch et al., 2020).

The general workflow of superimposing VSP or segmentations of critical structures on intraoperative fluoroscopic images could also be useful for other treatments. Superimposing structures or a VSP can be most valuable when fragile structures, e.g., neuro- or vascular structures, need to be avoided or in cases where it is difficult to translate the VSP to intraoperative fluoroscopic imaging. As a result, it could potentially reduce complication rates and enhance the accuracy and efficiency of procedures. Possible application areas are orthopedic, trauma, spine, or vascular surgery.

## 5 Conclusion

This study described a new method that provides additional intraoperative guidance to increase implant placement accuracy and avoid damaging critical structures. VSPs were successfully superimposed onto fluoroscopic images during five SIJF procedures, providing additional guidance during surgery. The current workflow was feasible for clinical use and resulted in acceptably low implant placement deviations. It was a valuable addition to SIJF and could potentially enhance implant placement accuracy. It could also be more cost-effective and time efficient than navigation-guided techniques or the conventional approach. However, these aspects, as well as further automation of the workflow, should be investigated in future comparative studies.

## Funding disclosures

All authors have no competing interests to declare that are relevant to the content of this article.

## Ethical approval

Ethical approval for retrospective and prospective analysis of VSP was obtained in July 2022 from the Research Ethic Committee of Medical Spectrum Twente (nWMO study, K22-24). The requirement for written informed consent was hereby waived for retrospective patients and prospective patients had to give written informed consent.

## Declaration of competing interest

The authors declare that they have no known competing financial interests or personal relationships that could have appeared to influence the work reported in this paper.
