# Supplementary material for: Sacroiliac joint fusion guided by intraoperatively superimposed virtual surgical planning using simulated fluoroscopic images
Source: Brain Spine. 2024 Aug 2;4:102905. doi: 10.1016/j.bas.2024.102905 (PMC11386036; doi:10.1016/j.bas.2024.102905)
Supplement: Multimedia component 1 [file mmc1.doc]

import tkinter
import tkinter.messagebox
from tkinter import ttk
import customtkinter
from tkinter import filedialog
import numpy as np
from PIL import Image, ImageTk, ImageOps, ImageDraw, ImageGrab
from Helperfunctions import procrustesfunc
import cv2
import os
from pptx.util import Cm
from pptx import Presentation
import pptx

customtkinter.set_appearance_mode("System") *# Modes: "System" (standard), "Dark", "Light"*customtkinter.set_default_color_theme("blue") *# Themes: "blue" (standard), "green", "dark-blue"*caseNumber = 0


*# create a popup gui to enter the patient reference number*def get_case_nr():
 global caseNumber
 caseNumber = entry1.get()
 root.destroy()


root = tkinter.Tk()
root.title('Fil in the patient reference number')
canvas1 = tkinter.Canvas(root, width=400, height=300)
canvas1.pack()
entry1 = tkinter.Entry(root)
canvas1.create_window(200, 140, window=entry1)
button1 = tkinter.Button(text='OK', command=get_case_nr)
canvas1.create_window(200, 180, window=button1)
root.mainloop()

print(f'Patient rerfernce number is: {caseNumber}')

*# Check of if directory for this patient exist in database*root_dir = os.path.abspath(r'ROOTPATIENTFILE')
root_dir_bestanden = os.path.abspath(r'ROOTHELPSCRIPTS')


patientDir = root_dir + r'\3DP-' + str(caseNumber)
Check = os.path.isdir(patientDir)

if Check == True:
 print(f"path found")
else:
 print(f"Error: path not found")
 raise SystemExit

*# Get dir path inside the Patient folder with all the preooperative calculations*patientScriptingDir = patientDir + r'/5 Scripting'
file = patientScriptingDir + r'/pt_nr.txt'
patientNumber = np.genfromtxt(file, dtype=str, encoding=None, delimiter=",")

*# Variables used to lead the implants in mimics*Implant = ["Craniaal", "Middel", "Caudaal"] *# Implant position names*ImplantNames = ["35", "40", "45", "50", "55", "60", "65", "70"] *# Implant lenghts in mm*currentImplant = np.genfromtxt(str(patientScriptingDir) + '\implantnames.txt', delimiter=",").astype(int) *# Load which inplant size is currently planned*lengths = ImplantNames[currentImplant[0]], ImplantNames[currentImplant[1]], ImplantNames[currentImplant[2]] *# Save implants*visualised_objectsPelvisOnly = []
visualised_objectsGuidepinsOnlyLAT = []
visualised_objectsGuidepinsOnlyInOut = []
visualised_objectsDonutsOnly = []
visualised_objectsDRRImplants = []


*# class for the intraoprative GUI*class App(customtkinter.CTk):
 def __init__(self):
 super().__init__()

 *# configure window* self.title("Intraopeartive GUI")
 self.geometry(f"{1370}x{830}")
 self.imageSize = 740

 *# configure grid layout (4x4)* self.grid_columnconfigure(1, weight=1)
 self.grid_columnconfigure((2, 3), weight=0)
 self.grid_rowconfigure((0, 1, 2), weight=1)
 self.side = "Not Yet loaded"
 self.driedpnummer = caseNumber
 self.patientScriptingDir = patientScriptingDir
 self.patientNumber = patientNumber
 self.LatImageNumberArray = np.zeros((21, 21))
 self.Blackimg = self.load_image(root_dir_bestanden + "\programFiles/Black.tif", invert=True)

 *# Create an array with the correct numbering for the 441 Lateral images* number = 1
 for i in range(21):
 for j in range(21):
 self.LatImageNumberArray[i, j] = number
 number += 1

 *# create sidebar frame with widgets* self.sidebar_frame = customtkinter.CTkFrame(self, width=140, corner_radius=0)
 self.sidebar_frame.grid(row=0, column=0, rowspan=4, sticky="nsew")
 self.sidebar_frame.grid_rowconfigure(7, weight=1)
 self.logo_label = customtkinter.CTkLabel(self.sidebar_frame, text="VSP Visualiser", font=customtkinter.CTkFont(size=20, weight="bold"))
 self.logo_label.grid(row=0, column=0, padx=20, pady=(20, 10))
 self.sidebar_button_1 = customtkinter.CTkButton(self.sidebar_frame, text="Load Patient", command=self.load_patient)
 self.sidebar_button_1.grid(row=1, column=0, padx=20, pady=10)
 self.sidebar_button_2 = customtkinter.CTkButton(self.sidebar_frame, text="Load Lat img", command=lambda: self.load_intraoperative_imageButtonFunc(0)) *# lateral* self.sidebar_button_2.grid(row=2, column=0, padx=20, pady=10)
 self.sidebar_button_3 = customtkinter.CTkButton(self.sidebar_frame, text="Load Inl img", command=lambda: self.load_intraoperative_imageButtonFunc(1))
 self.sidebar_button_3.grid(row=3, column=0, padx=20, pady=10)
 self.sidebar_button_4 = customtkinter.CTkButton(self.sidebar_frame, text="Load Outl img", command=lambda: self.load_intraoperative_imageButtonFunc(2))
 self.sidebar_button_4.grid(row=4, column=0, padx=20, pady=10)
 self.sidebar_button_5 = customtkinter.CTkButton(self.sidebar_frame, text="Make powerpoint", command=self.make_powerpoint)
 self.sidebar_button_5.grid(row=5, column=0, padx=20, pady=10)
 self.sidebar_button_6 = customtkinter.CTkButton(self.sidebar_frame, text="minimize", command=self.minimize)
 self.sidebar_button_6.grid(row=6, column=0, padx=20, pady=10)
 self.appearance_mode_label = customtkinter.CTkLabel(self.sidebar_frame, text="Appearance Mode:", anchor="w")
 self.appearance_mode_label.grid(row=8, column=0, padx=20, pady=(10, 0))
 self.appearance_mode_optionemenu = customtkinter.CTkOptionMenu(self.sidebar_frame, values=["Light", "Dark", "System"], command=self.change_appearance_mode_event)
 self.appearance_mode_optionemenu.grid(row=9, column=0, padx=20, pady=(10, 10))
 self.scaling_label = customtkinter.CTkLabel(self.sidebar_frame, text="UI Scaling:", anchor="w")
 self.scaling_label.grid(row=10, column=0, padx=20, pady=(10, 0))
 self.scaling_optionemenu = customtkinter.CTkOptionMenu(self.sidebar_frame, values=["80%", "90%", "100%", "110%", "120%"], command=self.change_scaling_event)
 self.scaling_optionemenu.grid(row=11, column=0, padx=20, pady=(10, 20))

 *# create main entry and button* self.topbar_frame = customtkinter.CTkFrame(self, height=20, corner_radius=5)
 self.topbar_frame.grid(row=0, column=1, padx=5, pady=5, sticky="nsew")
 self.number_label = customtkinter.CTkLabel(self.topbar_frame, text="Nummer: ", font=customtkinter.CTkFont(size=15, weight="bold"))
 self.number_label.grid(row=0, column=0, padx=20, pady=(10, 0))
 self.side_label = customtkinter.CTkLabel(self.topbar_frame, text="Kant: ", font=customtkinter.CTkFont(size=15, weight="bold"))
 self.side_label.grid(row=0, column=1, padx=20, pady=(10, 0))

 *# Create a tabview for each task in the GUI* self.tabview = customtkinter.CTkTabview(self, height=680)
 self.tabview.grid(row=1, column=1, padx=(10, 10), pady=(10, 10), sticky="nsew")
 self.tabview.add("Lat: Choose angle")
 self.tabview.add("Lat: Img registration")
 self.tabview.add("Inlet: Choose angle")
 self.tabview.add("Inlet: Img registration")
 self.tabview.add("Outlet: Choose angle")
 self.tabview.add("Outlet: Img registration")

 *# __________________________________ Start of tabview: Lat: Choose angle ______________________________________* self.main_frame_lat = customtkinter.CTkFrame(self.tabview.tab("Lat: Choose angle"), height=380, corner_radius=5)
 self.main_frame_lat.grid(row=0, column=0, rowspan=3, padx=5, pady=5, sticky="nsew")
 self.main_frame_lat.grid_rowconfigure(3, weight=1)

 *# Virtual Fluoroscopic image* self.canvasvirt = customtkinter.CTkCanvas(self.main_frame_lat, width=self.imageSize, height=self.imageSize, borderwidth=3, relief="sunken")
 self.canvasvirt.image = self.Blackimg
 self.image_containervirt = self.canvasvirt.create_image(self.imageSize / 2, self.imageSize / 2, image=self.Blackimg)
 self.canvasvirt.grid(row=0, column=1, padx=(10, 10), pady=(10, 10))

 *# create sliders* self.sliderHorizontal = customtkinter.CTkSlider(self.main_frame_lat, orientation="vertical", from_=0, to=20, command=self.changeDRRLateraal)
 self.sliderHorizontal.grid(row=0, column=0, rowspan=1, padx=(10, 10), pady=(10, 10), sticky="ns")
 self.sliderVertical = customtkinter.CTkSlider(self.main_frame_lat, from_=0, to=20, command=self.changeDRRLateraal)
 self.sliderVertical.grid(row=1, column=1, padx=(20, 10), pady=(10, 10), sticky="ew")

 *# intraoprative image* self.canvasreal = customtkinter.CTkCanvas(self.main_frame_lat, width=self.imageSize, height=self.imageSize, borderwidth=3, relief="sunken")
 self.canvasreal.image = self.Blackimg
 self.image_containerreal = self.canvasreal.create_image(self.imageSize / 2, self.imageSize / 2, image=self.Blackimg)
 self.canvasreal.grid(row=0, column=2, columnspan=2, padx=(10, 10), pady=(10, 10))
 self.slider_realImg = customtkinter.CTkSlider(self.main_frame_lat, from_=-125, to=125, number_of_steps=80,
 command=lambda val, view=0: self.rotate_real_imag(val, view))
 self.slider_realImg.grid(row=1, column=2, columnspan=2, padx=(20, 10), pady=(10, 10), sticky="ew")

 self.flipimagehorizontal = customtkinter.CTkImage(Image.open(root_dir_bestanden + "\programFiles/flip-6.png"), size=(30, 30))
 self.flipHorizontal = customtkinter.CTkButton(self.main_frame_lat, text="Flip", image=self.flipimagehorizontal, compound='left', command=lambda: self.flip("horizontal", 0))
 self.flipHorizontal.grid(row=2, column=2, padx=(5, 5), pady=(5, 5))

 self.flipimagevertical = customtkinter.CTkImage(Image.open(root_dir_bestanden + "\programFiles/flip-5.png"), size=(30, 30))
 self.flipVertical = customtkinter.CTkButton(self.main_frame_lat, text="Flip", image=self.flipimagevertical, compound='left', command=lambda: self.flip("vertical", 0))
 self.flipVertical.grid(row=2, column=3, padx=(5, 5), pady=(5, 5))
 *# __________________________________ End of tabview: Lat: Choose angle ______________________________________

 # ________________________________ Start of tabview: Lat: Img registration _____________________________________* self.RegistrationFrame = customtkinter.CTkFrame(self.tabview.tab("Lat: Img registration"), height=380, corner_radius=5)
 self.RegistrationFrame.grid(row=0, column=0, rowspan=3, padx=5, pady=5, sticky="nsew")
 self.RegistrationFrame.grid_rowconfigure(5, weight=1)

 *# Virtual Fluoroscopic image* self.canvasvirtTwo = customtkinter.CTkCanvas(self.RegistrationFrame, width=self.imageSize, height=self.imageSize, borderwidth=3, relief="sunken")
 self.canvasvirtTwo.image = self.Blackimg
 self.image_containervirtTwo = self.canvasvirtTwo.create_image(self.imageSize / 2, self.imageSize / 2, image=self.Blackimg)
 self.canvasvirtTwo.grid(row=0, column=0, columnspan=3, padx=(10, 10), pady=(10, 10))

 *# intraoprative image* self.canvasRealTwo = customtkinter.CTkCanvas(self.RegistrationFrame, width=self.imageSize, height=self.imageSize, borderwidth=3, relief="sunken")
 self.canvasRealTwo.image = self.Blackimg
 self.image_containerrealTwo = self.canvasRealTwo.create_image(self.imageSize / 2, self.imageSize / 2, image=self.Blackimg)
 self.canvasRealTwo.grid(row=0, column=3, columnspan=3, padx=(10, 10), pady=(10, 10))

 *# Create all the buttons* self.laadbutton = customtkinter.CTkButton(self.RegistrationFrame, text="Load images", command=lambda: self.load_images_new_tab(0))
 self.InputLabel = customtkinter.CTkLabel(self.RegistrationFrame, text="Select 8 points and press on: ")
 self.procrustbtn = customtkinter.CTkButton(self.RegistrationFrame, text="Run Image Registration", command=lambda: self.registerpoints(0))
 self.undobtn_real = customtkinter.CTkButton(self.RegistrationFrame, text="Undo last point real", command=lambda: self.undo_click(0, 0), width=50)
 self.undobtn_virt = customtkinter.CTkButton(self.RegistrationFrame, text="Undo last point virtual", command=lambda: self.undo_click(0, 1), width=50)
 self.undobtn_all = customtkinter.CTkButton(self.RegistrationFrame, text="Undo all points", command=lambda: self.undo_all(0), width=50)

 self.laadbutton.grid(row=1, column=0, padx=(10, 10), pady=1, sticky='w')
 self.InputLabel.grid(row=2, column=0, columnspan=2, padx=(10, 10), sticky='w')
 self.procrustbtn.grid(row=3, column=0, padx=(10, 10), pady=1, sticky='w')
 self.undobtn_real.grid(row=1, column=1, padx=(10, 10), pady=1, sticky='w')
 self.undobtn_virt.grid(row=2, column=1, padx=(10, 10), pady=1, sticky='w')
 self.undobtn_all.grid(row=3, column=1, padx=(10, 10), pady=1, sticky='w')

 self.hide_all_btn = customtkinter.CTkSwitch(master=self.RegistrationFrame, text="Hide all text", command=lambda: self.hide_all(0))
 self.hide_all_btn.grid(row=1, column=3, padx=(10, 10), pady=1, sticky='w')
 self.hideDonuts_btn = customtkinter.CTkSwitch(master=self.RegistrationFrame, text="Hide donuts", command=lambda: self.hideDonutsOrPins(0))
 self.hideDonuts_btn.grid(row=2, column=3, padx=(10, 10), pady=1, sticky='w')
 self.hidePins_btn = customtkinter.CTkSwitch(master=self.RegistrationFrame, text="Hide Pins", command=lambda: self.hideDonutsOrPins(0))
 self.hidePins_btn.grid(row=3, column=3, padx=(10, 10), pady=1, sticky='w')

 self.canvasvirtTwo.bind('<Button-1>', lambda val, view=0: self.onmouse_virt(val, view))
 self.canvasRealTwo.bind('<Button-1>', lambda val, view=0: self.onmouse_real(val, view))
 self.coordinatesReal = []
 self.coordinatesVirt = []
 *# __________________________________ End of tabview: Lat: Choose angle ______________________________________

 #
 # INLET
 #

 # ________________________________ Start of tabview: Inlet: Choose angle _____________________________________* self.main_frame_Inlet = customtkinter.CTkFrame(self.tabview.tab("Inlet: Choose angle"), height=380, corner_radius=5)
 self.main_frame_Inlet.grid(row=0, column=0, rowspan=3, padx=5, pady=5, sticky="nsew")
 self.main_frame_Inlet.grid_rowconfigure(3, weight=1)

 *# Virtual Fluoroscopic image* self.canvasvirtInlet = customtkinter.CTkCanvas(self.main_frame_Inlet, width=self.imageSize, height=self.imageSize, borderwidth=3, relief="sunken")
 self.canvasvirtInlet.image = self.Blackimg
 self.image_containervirtInlet = self.canvasvirtInlet.create_image(self.imageSize / 2, self.imageSize / 2, image=self.Blackimg)
 self.canvasvirtInlet.grid(row=0, column=1, padx=(10, 10), pady=(10, 10))
 self.sliderHorizontalInlet = customtkinter.CTkSlider(self.main_frame_Inlet, orientation="vertical", from_=1, to=21, command=lambda val, view=1: self.changeDRRInletOutlet(val, view))
 self.sliderHorizontalInlet.grid(row=0, column=0, rowspan=1, padx=(10, 10), pady=(10, 10), sticky="ns")

 *# intraoprative image* self.canvasrealInlet = customtkinter.CTkCanvas(self.main_frame_Inlet, width=self.imageSize, height=self.imageSize, borderwidth=3, relief="sunken")
 self.canvasrealInlet.image = self.Blackimg
 self.image_containerrealInlet = self.canvasrealInlet.create_image(self.imageSize / 2, self.imageSize / 2, image=self.Blackimg)
 self.canvasrealInlet.grid(row=0, column=2, columnspan=2, padx=(10, 10), pady=(10, 10))
 self.slider_realImgInlet = customtkinter.CTkSlider(self.main_frame_Inlet, from_=-125, to=125, number_of_steps=80, command=lambda val, view=1: self.rotate_real_imag(val, view))
 self.slider_realImgInlet.grid(row=1, column=2, columnspan=2, padx=(20, 10), pady=(10, 10), sticky="ew")

 self.flipimagehorizontal = customtkinter.CTkImage(Image.open(root_dir_bestanden + "\programFiles/flip-6.png"), size=(30, 30))
 self.flipHorizontal = customtkinter.CTkButton(self.main_frame_Inlet, text="Flip", image=self.flipimagehorizontal, compound='left', command=lambda: self.flip("vertical", 1))
 self.flipHorizontal.grid(row=2, column=2, padx=(5, 5), pady=(5, 5))

 self.flipimagevertical = customtkinter.CTkImage(Image.open(root_dir_bestanden + "\programFiles/flip-5.png"), size=(30, 30))
 self.flipVertical = customtkinter.CTkButton(self.main_frame_Inlet, text="Flip", image=self.flipimagevertical, compound='left', command=lambda: self.flip("horizontal", 1))
 self.flipVertical.grid(row=2, column=3, padx=(5, 5), pady=(5, 5))
 *# __________________________________ End of tabview: Inlet: Choose angle ______________________________________

 # ________________________________ Start of tabview: Inlet: Img registration _____________________________________* self.RegistrationFrameInlet = customtkinter.CTkFrame(self.tabview.tab("Inlet: Img registration"), height=380, corner_radius=5)
 self.RegistrationFrameInlet.grid(row=0, column=0, rowspan=3, padx=5, pady=5, sticky="nsew")
 self.RegistrationFrameInlet.grid_rowconfigure(5, weight=1)

 *# Virtual Fluoroscopic image* self.canvasvirtTwoInlet = customtkinter.CTkCanvas(self.RegistrationFrameInlet, width=self.imageSize, height=self.imageSize, borderwidth=3, relief="sunken")
 self.canvasvirtTwoInlet.image = self.Blackimg
 self.image_containervirtTwoInlet = self.canvasvirtTwoInlet.create_image(self.imageSize / 2, self.imageSize / 2, image=self.Blackimg)
 self.canvasvirtTwoInlet.grid(row=0, column=0, columnspan=3, padx=(10, 10), pady=(10, 10))

 *# intraoprative image* self.canvasRealTwoInlet = customtkinter.CTkCanvas(self.RegistrationFrameInlet, width=self.imageSize, height=self.imageSize, borderwidth=3, relief="sunken")
 self.canvasRealTwoInlet.image = self.Blackimg
 self.image_containerrealTwoInlet = self.canvasRealTwoInlet.create_image(self.imageSize / 2, self.imageSize / 2, image=self.Blackimg)
 self.canvasRealTwoInlet.grid(row=0, column=3, columnspan=3, padx=(10, 10), pady=(10, 10))

 *# buttons* self.laadbuttonInlet = customtkinter.CTkButton(self.RegistrationFrameInlet, text="Load images", command=lambda: self.load_images_new_tab(1))
 self.InputLabelInlet = customtkinter.CTkLabel(self.RegistrationFrameInlet, text="Select 8 points and press on: ")
 self.procrustbtnInlet = customtkinter.CTkButton(self.RegistrationFrameInlet, text="Run Image Registration", command=lambda: self.registerpoints(1))
 self.undobtn_realInlet = customtkinter.CTkButton(self.RegistrationFrameInlet, text="Undo last point real", command=lambda: self.undo_click(1, 0), width=50)
 self.undobtn_virtInlet = customtkinter.CTkButton(self.RegistrationFrameInlet, text="Undo last point virtual", command=lambda: self.undo_click(1, 1), width=50)
 self.undobtn_allInlet = customtkinter.CTkButton(self.RegistrationFrameInlet, text="Undo all points", command=lambda: self.undo_all(1), width=50)

 self.laadbuttonInlet.grid(row=1, column=0, padx=(10, 10), pady=1, sticky='w')
 self.InputLabelInlet.grid(row=2, column=0, columnspan=2, padx=(10, 10), sticky='w')
 self.procrustbtnInlet.grid(row=3, column=0, padx=(10, 10), pady=1, sticky='w')
 self.undobtn_realInlet.grid(row=1, column=1, padx=(10, 10), pady=1, sticky='w')
 self.undobtn_virtInlet.grid(row=2, column=1, padx=(10, 10), pady=1, sticky='w')
 self.undobtn_allInlet.grid(row=3, column=1, padx=(10, 10), pady=1, sticky='w')

 self.hide_all_btnInlet = customtkinter.CTkSwitch(master=self.RegistrationFrameInlet, text="Hide all text", command=lambda: self.hide_all(1))
 self.hide_all_btnInlet.grid(row=1, column=3, padx=(10, 10), pady=1, sticky='w')
 self.hideDonuts_btnInlet = customtkinter.CTkSwitch(master=self.RegistrationFrameInlet, text="Hide Implants", command=lambda: self.hideDonutsOrPins(1))
 self.hideDonuts_btnInlet.grid(row=2, column=3, padx=(10, 10), pady=1, sticky='w')
 self.hidePins_btnInlet = customtkinter.CTkSwitch(master=self.RegistrationFrameInlet, text="Hide Pins", command=lambda: self.hideDonutsOrPins(1))
 self.hidePins_btnInlet.grid(row=3, column=3, padx=(10, 10), pady=1, sticky='w')

 *# Bind the left button click to the function so the user can click point in each image* self.canvasvirtTwoInlet.bind('<Button-1>', lambda val, view=1: self.onmouse_virt(val, view))
 self.canvasRealTwoInlet.bind('<Button-1>', lambda val, view=1: self.onmouse_real(val, view))
 *# Array to save the coordinates* self.coordinatesRealInlet = []
 self.coordinatesVirtInlet = []
 *# __________________________________ End of tabview: Inlet: Img registration ______________________________________

 #
 # OUTLET
 #

 # ________________________________ Start of tabview: Outlet: Choose angle _____________________________________
 # create the Lateral tav tabview 1* self.main_frame_Outlet = customtkinter.CTkFrame(self.tabview.tab("Outlet: Choose angle"), height=380, corner_radius=5)
 self.main_frame_Outlet.grid(row=0, column=0, rowspan=3, padx=5, pady=5, sticky="nsew")
 self.main_frame_Outlet.grid_rowconfigure(3, weight=1)

 *# Virtual Fluoroscopic image* self.canvasvirtOutlet = customtkinter.CTkCanvas(self.main_frame_Outlet, width=self.imageSize, height=self.imageSize, borderwidth=3, relief="sunken")
 self.canvasvirtOutlet.image = self.Blackimg
 self.image_containervirtOutlet = self.canvasvirtOutlet.create_image(self.imageSize / 2, self.imageSize / 2, image=self.Blackimg)
 self.canvasvirtOutlet.grid(row=0, column=1, padx=(10, 10), pady=(10, 10))
 self.sliderHorizontalOutlet = customtkinter.CTkSlider(self.main_frame_Outlet, orientation="vertical", from_=1, to=21, command=lambda val, view=2: self.changeDRRInletOutlet(val, view))
 self.sliderHorizontalOutlet.grid(row=0, column=0, rowspan=1, padx=(10, 10), pady=(10, 10), sticky="ns")

 *# intraoprative image* self.canvasrealOutlet = customtkinter.CTkCanvas(self.main_frame_Outlet, width=self.imageSize, height=self.imageSize, borderwidth=3, relief="sunken")
 self.canvasrealOutlet.image = self.Blackimg
 self.image_containerrealOutlet = self.canvasrealOutlet.create_image(self.imageSize / 2, self.imageSize / 2, image=self.Blackimg)
 self.canvasrealOutlet.grid(row=0, column=2, columnspan=2, padx=(10, 10), pady=(10, 10))
 self.slider_realImgOutlet = customtkinter.CTkSlider(self.main_frame_Outlet, from_=-125, to=125, number_of_steps=80, command=lambda val, view=2: self.rotate_real_imag(val, view))
 self.slider_realImgOutlet.grid(row=1, column=2, columnspan=2, padx=(20, 10), pady=(10, 10), sticky="ew")

 *# buttons* self.flipimagehorizontal = customtkinter.CTkImage(Image.open(root_dir_bestanden + "\programFiles/flip-6.png"), size=(30, 30))
 self.flipHorizontal = customtkinter.CTkButton(self.main_frame_Outlet, text="Flip", image=self.flipimagehorizontal, compound='left', command=lambda: self.flip("vertical", 2))
 self.flipHorizontal.grid(row=2, column=2, padx=(5, 5), pady=(5, 5))
 self.flipimagevertical = customtkinter.CTkImage(Image.open(root_dir_bestanden + "\programFiles/flip-5.png"), size=(30, 30))
 self.flipVertical = customtkinter.CTkButton(self.main_frame_Outlet, text="Flip", image=self.flipimagevertical, compound='left', command=lambda: self.flip("horizontal", 2))
 self.flipVertical.grid(row=2, column=3, padx=(5, 5), pady=(5, 5))

 self.bind("<Up>", lambda e: self.keys(e))
 self.bind("<Down>", lambda e: self.keys(e))
 self.bind("<Left>", lambda e: self.keys(e))
 self.bind("<Right>", lambda e: self.keys(e))
 *# __________________________________ End of tabview: Outlet: Choose angle ______________________________________

 # ________________________________ Start of tabview: Outlet: Img registration _____________________________________* self.RegistrationFrameOutlet = customtkinter.CTkFrame(self.tabview.tab("Outlet: Img registration"), height=380, corner_radius=5)
 self.RegistrationFrameOutlet.grid(row=0, column=0, rowspan=3, padx=5, pady=5, sticky="nsew")
 self.RegistrationFrameOutlet.grid_rowconfigure(5, weight=1)

 *# Virtual Fluoroscopic image* self.canvasvirtTwoOutlet = customtkinter.CTkCanvas(self.RegistrationFrameOutlet, width=self.imageSize, height=self.imageSize, borderwidth=3, relief="sunken")
 self.canvasvirtTwoOutlet.image = self.Blackimg
 self.image_containervirtTwoOutlet = self.canvasvirtTwoOutlet.create_image(self.imageSize / 2, self.imageSize / 2, image=self.Blackimg)
 self.canvasvirtTwoOutlet.grid(row=0, column=0, columnspan=3, padx=(10, 10), pady=(10, 10))

 *# intraoprative image* self.canvasRealTwoOutlet = customtkinter.CTkCanvas(self.RegistrationFrameOutlet, width=self.imageSize, height=self.imageSize, borderwidth=3, relief="sunken")
 self.canvasRealTwoOutlet.image = self.Blackimg
 self.image_containerrealTwoOutlet = self.canvasRealTwoOutlet.create_image(self.imageSize / 2, self.imageSize / 2, image=self.Blackimg)
 self.canvasRealTwoOutlet.grid(row=0, column=3, columnspan=3, padx=(10, 10), pady=(10, 10))

 *# buttons* self.laadbuttonOutlet = customtkinter.CTkButton(self.RegistrationFrameOutlet, text="Load images", command=lambda: self.load_images_new_tab(2))
 self.InputLabelOutlet = customtkinter.CTkLabel(self.RegistrationFrameOutlet, text="Select 8 points and press on: ")
 self.procrustbtnOutlet = customtkinter.CTkButton(self.RegistrationFrameOutlet, text="Run Image Registration", command=lambda: self.registerpoints(2))
 self.undobtn_realOutlet = customtkinter.CTkButton(self.RegistrationFrameOutlet, text="Undo last point real", command=lambda: self.undo_click(2, 0), width=50)
 self.undobtn_virtOutlet = customtkinter.CTkButton(self.RegistrationFrameOutlet, text="Undo last point virtual", command=lambda: self.undo_click(2, 1), width=50)
 self.undobtn_allOutlet = customtkinter.CTkButton(self.RegistrationFrameOutlet, text="Undo all points", command=lambda: self.undo_all(2), width=50)

 self.laadbuttonOutlet.grid(row=1, column=0, padx=(10, 10), pady=1, sticky='w')
 self.InputLabelOutlet.grid(row=2, column=0, columnspan=2, padx=(10, 10), sticky='w')
 self.procrustbtnOutlet.grid(row=3, column=0, padx=(10, 10), pady=1, sticky='w')
 self.undobtn_realOutlet.grid(row=1, column=1, padx=(10, 10), pady=1, sticky='w')
 self.undobtn_virtOutlet.grid(row=2, column=1, padx=(10, 10), pady=1, sticky='w')
 self.undobtn_allOutlet.grid(row=3, column=1, padx=(10, 10), pady=1, sticky='w')

 self.hide_all_btnOutlet = customtkinter.CTkSwitch(master=self.RegistrationFrameOutlet, text="Hide all text", command=lambda: self.hide_all(2))
 self.hide_all_btnOutlet.grid(row=1, column=3, padx=(10, 10), pady=1, sticky='w')
 self.hideDonuts_btnOutlet = customtkinter.CTkSwitch(master=self.RegistrationFrameOutlet, text="Hide Implants", command=lambda: self.hideDonutsOrPins(2))
 self.hideDonuts_btnOutlet.grid(row=2, column=3, padx=(10, 10), pady=1, sticky='w')
 self.hidePins_btnOutlet = customtkinter.CTkSwitch(master=self.RegistrationFrameOutlet, text="Hide Pins", command=lambda: self.hideDonutsOrPins(2))
 self.hidePins_btnOutlet.grid(row=3, column=3, padx=(10, 10), pady=1, sticky='w')

 self.canvasvirtTwoOutlet.bind('<Button-1>', lambda val, view=2: self.onmouse_virt(val, view))
 self.canvasRealTwoOutlet.bind('<Button-1>', lambda val, view=2: self.onmouse_real(val, view))
 self.coordinatesRealOutlet = []
 self.coordinatesVirtOutlet = []
 *# ________________________________ End of tabview: Outlet: Img registration _____________________________________* self.attributes('-fullscreen', True)
 *# Initiate all arrays
 #
 #* self.AllDirectoryNAmes = ['/LatImageArray/', '/InletImageArray/', '/OutletImageArray/']
 self.AllintratopImg = [[], [], []]
 self.AllhorizontalFlip = [False, False, False]
 self.AllverticalFlip = [False, False, False]
 self.AllrotateAngle = [None, None, None]
 self.Allcrop = [False, True, True]
 self.AllCanvasreal = [self.canvasreal, self.canvasrealInlet, self.canvasrealOutlet]
 self.AllImage_containerreal = [self.image_containerreal, self.image_containerrealInlet, self.image_containerrealOutlet]
 self.AllImage_containerVirt = [self.image_containervirt, self.image_containervirtInlet, self.image_containervirtOutlet]
 self.Allcanvasvirt = [self.canvasvirt, self.canvasvirtInlet, self.canvasvirtOutlet]
 self.AllcanvasRealTwo = [self.canvasRealTwo, self.canvasRealTwoInlet, self.canvasRealTwoOutlet]
 self.AllcanvasvirtTwo = [self.canvasvirtTwo, self.canvasvirtTwoInlet, self.canvasvirtTwoOutlet]
 self.Allimage_containerrealTwo = [self.image_containerrealTwo, self.image_containerrealTwoInlet, self.image_containerrealTwoOutlet]
 self.Allimage_containervirtTwo = [self.image_containervirtTwo, self.image_containervirtTwoInlet, self.image_containervirtTwoOutlet]
 self.AllcoordinatesReal = [self.coordinatesReal, self.coordinatesRealInlet, self.coordinatesRealOutlet]
 self.AllcoordinatesVirt = [self.coordinatesVirt, self.coordinatesVirtInlet, self.coordinatesVirtOutlet]
 self.Allhide_all_btn = [self.hide_all_btn, self.hide_all_btnInlet, self.hide_all_btnOutlet]
 self.AllhideDonuts_btn = [self.hideDonuts_btn, self.hideDonuts_btnInlet, self.hideDonuts_btnOutlet]
 self.AllhidePins_btn = [self.hidePins_btn, self.hidePins_btnInlet, self.hidePins_btnOutlet]
 self.AllsliderHorizontal = [self.sliderHorizontal, self.sliderHorizontalInlet, self.sliderHorizontalOutlet]
 self.AllRegistrationframes = [self.RegistrationFrame, self.RegistrationFrameInlet, self.RegistrationFrameOutlet]
 self.AllImg = [[], [], []] *# real images* self.AllCurrentDRR = [[], [], []]
 self.AllRegisterdPins = [[], [], []]
 self.AllRegisterdDonuts = [[], [], []]
 self.AllRegisterdTogether = [[], [], []]
 self.AllcanvasRealTwoDonutsOrPins = [[], [], []]
 self.AllcanvasvirtTwoDonutsOrPins = [[], [], []]
 self.AllPins = [[], [], []]
 self.AllDonuts = [[], [], []]
 self.AllTogether = [[], [], []]
 self.FluoroscopicViews = [[], [], []]
 self.AllImgNonphoto = [[], [], []] *# real images but not photoimages* self.images = [[[], []], [[], []], [[], []]] *# images for in the powerpoint* self.AllImageNames = ["Lateraal", "Inlet", "Outlet"]

 self.DRRNamesSingle = ["Guidepinsonly", "Donutsonly"]
 self.AllobjectsLAt = [visualised_objectsGuidepinsOnlyLAT, visualised_objectsDonutsOnly]
 self.AllobjectsInOut = [visualised_objectsGuidepinsOnlyInOut, visualised_objectsDRRImplants]

 def keys(self, e):
 *"""
 The function `keys` is used to handle key events and update the values of sliders based on the current tab and
 direction of the key press and change the simulated image.

 :param e: the event of the keypress by the user
 """* currentTab = self.tabview.get()
 if currentTab == "Lat: Choose angle":
 view = 0
 elif currentTab == "Inlet: Choose angle":
 view = 1
 elif currentTab == "Outlet: Choose angle":
 view = 2
 else:
 return
 direction = e.keysym
 if direction == "Up":
 self.AllsliderHorizontal[view].set(self.AllsliderHorizontal[view].get() + 1)
 elif direction == "Down":
 self.AllsliderHorizontal[view].set(self.AllsliderHorizontal[view].get() - 1)
 elif direction == "Left" and view == 0:
 self.sliderVertical.set(self.sliderVertical.get() - 1)
 elif direction == "Right" and view == 0:
 self.sliderVertical.set(self.sliderVertical.get() + 1)
 if view == 0:
 self.changeDRRLateraal(e)
 else:
 self.changeDRRInletOutlet(e, view)

 def change_appearance_mode_event(self, new_appearance_mode: str):
 *"""
 The function `change_appearance_mode_event` sets the appearance mode of a custom tkinter module.
 """* customtkinter.set_appearance_mode(new_appearance_mode)

 def change_scaling_event(self, new_scaling: str):
 *"""
 The function `change_scaling_event` converts a string representing a percentage to a float and then calls a function
 to set the scaling of a widget.

 """* new_scaling_float = int(new_scaling.replace("%", "")) / 100
 customtkinter.set_widget_scaling(new_scaling_float)

 def load_patient(self):
 *"""
 The function loads patient information and images into a GUI interface.
 """* self.scriptingDir = self.patientScriptingDir
 file = self.scriptingDir + r'/Pt_nr.txt'
 self.patientnumber = np.genfromtxt(file, dtype=str, encoding=None, delimiter=",")
 file = self.scriptingDir + r'/Side.txt'
 self.side = np.genfromtxt(file, dtype=str, encoding=None, delimiter=",")

 self.number_label.configure(text=f'Nummer: DEMO')
 self.side_label.configure(text=f'Kant: : {self.side}')

 startingImagesNames = ['221', '11', '11'] *# These are the center images* self.rotate = [True, False, False] *# by default the lateral image is rotated* self.flipimg = [True, True, True] *# by default all images are flipped* for i in range(3):
 imagename = self.scriptingDir + self.AllDirectoryNAmes[i] + startingImagesNames[i] + r'.bmp'
 img = self.load_image(imagename, invert=False, crop=self.Allcrop[i], rotate=self.rotate[i], flip=self.flipimg[i])
 self.Allcanvasvirt[i].itemconfig(self.AllImage_containerVirt[i], image=img)
 self.Allcanvasvirt[i].image = img

 def load_image(self, img, invert=False, crop=False, photo_img=True, rotate=False, flip=False):
 *"""
 The function takes an image file as input and performs various image processing operations such as
 resizing, cropping, inverting, rotating, and flipping, and returns the processed image. The function is called each
 time the virtual fluoroscopic image is changed

 :param img: The "img" parameter is the path or file object of the image that you want to load.
 :param invert: The "invert" parameter determines whether the image should be inverted or not.
 :param crop: The "crop" parameter determines whether or not to crop the image.
 :param photo_img: The `photo_img` parameter is a boolean flag that determines whether the loaded image should be
 converted to a `PhotoImage` object. This is needed for some image visualisation inside the gui
 :param rotate: The "rotate" parameter determines whether or not to rotate the image.
 :param flip: The "flip" parameter determines whether the image should be flipped horizontally.
 :return: the processed image.
 """* img = Image.open(img)
 if crop:
 cropsize = 500
 else:
 cropsize = 700
 img = img.resize((self.imageSize + cropsize, self.imageSize + cropsize), Image.Resampling.LANCZOS)
 img = img.crop((cropsize / 2, cropsize / 2, self.imageSize + cropsize / 2, self.imageSize + cropsize / 2))
 if invert:
 img = ImageOps.invert(img)
 img = img.resize((self.imageSize, self.imageSize), Image.Resampling.LANCZOS)
 if rotate:
 if self.side == "Links":
 img = img.rotate(-90)
 if self.side == "Rechts":
 img = img.rotate(90)
 if flip:
 img = img.transpose(Image.Transpose.FLIP_LEFT_RIGHT)
 if photo_img:
 img = ImageTk.PhotoImage(img)
 return img

 def load_intraoperative_imageButtonFunc(self, view):
 *"""
 The function loads an intraoperative image, converts it to grayscale, and calls the load intraop img function.

 :param view: The "view" parameter is used to specify the current view in the GUI.
 """* self.intraoperativeAfbeeldingName = filedialog.askopenfilename(title='Open intraoperatieve afbeelding')
 CarmImage = False
 if CarmImage:
 img = Image.open(self.intraoperativeAfbeeldingName)
 img = np.array(img)
 img = (img / np.max(img)) * 255
 img = np.int8(img)
 else:
 img = Image.open(self.intraoperativeAfbeeldingName)
 img = np.array(img)

 self.AllintratopImg[view] = Image.fromarray(img)
 self.AllintratopImg[view].convert("L")
 self.load_intraop_image(self.AllintratopImg[view], view, invert=False)

 def load_intraop_image(self, img, view, invert=False):
 *"""
 The function takes an image, applies various transformations to it based on the view, and
 updates the image displayed on a canvas.

 :param img: The `img` parameter is an image object for the intraoperative image.
 :param invert: The `invert` parameter is a boolean flag that determines whether the image should be inverted or not.
 """* if invert:
 img = ImageOps.invert(img)
 if self.AllhorizontalFlip[view]:
 img = img.transpose(Image.Transpose.FLIP_TOP_BOTTOM)
 if self.AllverticalFlip[view]:
 img = img.transpose(Image.Transpose.FLIP_LEFT_RIGHT)
 if self.AllrotateAngle[view]:
 img = img.rotate(self.AllrotateAngle[view], fillcolor='white')
 img = img.resize((self.imageSize, self.imageSize), Image.Resampling.LANCZOS)
 self.AllImgNonphoto[view] = img
 self.AllImg[view] = ImageTk.PhotoImage(img)
 self.AllCanvasreal[view].itemconfig(self.AllImage_containerreal[view], image=self.AllImg[view])
 self.AllCanvasreal[view].image = self.AllImg[view]

 def rotate_real_imag(self, angle, view):
 *"""
 The function saves the rotation angle for the image in the correct view and loads the reloads the image

 """* self.AllrotateAngle[view] = angle
 self.load_intraop_image(self.AllintratopImg[view], view)

 def flip(self, direction, view):
 *"""
 The function saves the flip state of the image and reloads the image

 """* if direction == "horizontal":
 self.AllhorizontalFlip[view] = not self.AllhorizontalFlip[view]
 if direction == "vertical":
 self.AllverticalFlip[view] = not self.AllverticalFlip[view]
 self.load_intraop_image(self.AllintratopImg[view], view)

 def changeDRRLateraal(self, event, view=0):
 *"""
 The function is called when the "angle" of the lateral simulated image is changed in the view witch the buttons or
 with the arrow keys.

 """* Horizontal = int(self.sliderHorizontal.get())
 Vertical = int(self.sliderVertical.get())
 imgnumber = int(self.LatImageNumberArray[Vertical, Horizontal])
 latImage = self.scriptingDir + self.AllDirectoryNAmes[view] + str(imgnumber) + r'.bmp'
 img = self.load_image(latImage, invert=False, crop=self.Allcrop[view], rotate=True, flip=self.flipimg[view])
 self.Allcanvasvirt[view].itemconfig(self.image_containervirt, image=img)
 self.Allcanvasvirt[view].image = img
 self.AllCurrentDRR[view] = img
 print(f"horizontal: {Horizontal}, vertical: {Vertical}")
 print(f"image number is {imgnumber}")

 def changeDRRInletOutlet(self, event, view):
 *"""
 The function is called when the "angle" of the inlet or outlet simulated image is changed in the view witch the buttons or
 with the arrow keys.

 """* Horizontal = int(self.AllsliderHorizontal[view].get())
 InletOutletImage = self.scriptingDir + self.AllDirectoryNAmes[view] + str(Horizontal) + r'.bmp'
 img = self.load_image(InletOutletImage, invert=False, crop=True, flip=self.flipimg[view])
 self.Allcanvasvirt[view].itemconfig(self.image_containervirt, image=img)
 self.Allcanvasvirt[view].image = img
 self.AllCurrentDRR[view] = img
 print(f"image number is {Horizontal}")

 *# Functions for registration tabs* def load_images_new_tab(self, view):
 *"""
 The function is called when the registration tab is initalized and the load img button is pressed. Based on the
 choosen parameters a fluoroscopic view is created in Mimics and two images are created with different items visiable


 """* self.AllcanvasRealTwo[view].itemconfig(self.Allimage_containerrealTwo[view], image=self.AllImg[view])
 self.AllcanvasRealTwo[view].image = self.AllImg[view]
 self.AllcanvasvirtTwo[view].itemconfig(self.Allimage_containervirtTwo[view], image=self.AllCurrentDRR[view])
 self.AllcanvasvirtTwo[view].image = self.AllCurrentDRR[view]
 self.FluoroscopicViews[view].attenuation_coefficient = 0

 Angles = np.genfromtxt(self.patientScriptingDir + '\Trueangles.txt', delimiter=",") *# the planned angles are loaded* Anglesview = Angles[view]
 if view == 0:
 Horizontal = (10 - int(self.sliderHorizontal.get())) * 0.5
 Vertical = (10 - int(self.sliderVertical.get())) * 0.5
 else:
 Vertical = (11 - int(self.AllsliderHorizontal[view].get())) * 1
 Horizontal = 0
 *# The planned angles and the user defined angles are used to determine the final angle of the image* print(f"vertical angle differnce is {Vertical}, Horizontal angle difference is {Horizontal}")
 self.FluoroscopicViews[view].lao_rao_angle = Anglesview[0] - Horizontal
 self.FluoroscopicViews[view].cran_caud_angle = Anglesview[1] - Vertical

 if view == 0:
 for i in range(2):
 name = str(self.AllImageNames[view] + "_" + self.DRRNamesSingle[i])
 self.export_view(self.FluoroscopicViews[view], self.AllobjectsLAt[i], name, imageSize=1200)
 else:
 for i in range(2):
 name = str(self.AllImageNames[view] + "_" + self.DRRNamesSingle[i])
 self.export_view(self.FluoroscopicViews[view], self.AllobjectsInOut[i], name, imageSize=1200)

 def onmouse_real(self, event, view):
 *"""
 The function appends the coordinates of a mouse event to a list and then calls another function to draw the
 coordinates on the intraopartive image canvas.
 """* self.AllcoordinatesReal[view].append((event.x, event.y))
 self.draw(self.AllcoordinatesReal[view], self.AllcanvasRealTwo[view])

 def onmouse_virt(self, event, view):
 *"""
 The function appends the coordinates of a mouse event to a list and then calls another function to draw the
 coordinates on the simulated fluoroscopy image canvas.
 """* self.AllcoordinatesVirt[view].append((event.x, event.y))
 self.draw(self.AllcoordinatesVirt[view], self.AllcanvasvirtTwo[view])

 def draw(self, coordinates, canvas):
 *"""
 The function takes a list of coordinates and a canvas object, and then draws text on the canvas at each coordinate
 with a number label. The offset may be depended on the screensize.
 """* for i in range(len(coordinates)):
 [x, y] = coordinates[i]
 print(f"x is : {x}, y is :{y}")
 canvas.create_text((x + 5), (y), fill='red', font=('Helvetica 15 bold'),
 text="> " + str(i + 1), tag="tag")

 def undo_click(self, view, canvas):
 *"""
 The `undo_click` function allows the user to undo the last click on a canvas by deleting the last set of coordinates
 and redrawing the image.
 """* if canvas == 0:
 if len(self.AllcoordinatesReal[view]) > 0:
 self.AllcanvasRealTwo[view].delete("tag")
 self.AllcoordinatesReal[view].pop()
 self.draw(self.AllcoordinatesReal[view], self.AllcanvasRealTwo[view])
 else:
 print("No points selected image are drawn yet")
 if canvas == 1:
 if len(self.AllcoordinatesVirt[view]) > 0:
 self.AllcanvasvirtTwo[view].delete("tag")
 self.AllcoordinatesVirt[view].pop()
 self.draw(self.AllcoordinatesVirt[view], self.AllcanvasvirtTwo[view])
 else:
 print("No points selected image are drawn yet")

 def undo_all(self, view):
 *"""
 The `undo_all` function deletes all points drawn on a canvas and clears the list of coordinates for both real and
 virtual images.
 """* if len(self.AllcoordinatesReal[view]) > 0:
 self.AllCanvasreal[view].delete("tag")
 self.AllcoordinatesReal[view] = []
 else:
 print("No points selected image are drawn yet")
 if len(self.AllcoordinatesVirt[view]) > 0:
 self.Allcanvasvirt[view].delete("tag")
 self.AllcoordinatesVirt[view] = []
 else:
 print("No points selected image are drawn yet")

 def hide_all(self, view):
 *"""
 The function `hide_all` checks the state of a button and either deletes all items with a specific tag from two
 canvas objects or redraws items on the canvas objects based on coordinates.

 """* state = self.Allhide_all_btn[view].get()
 if state == 1:
 self.AllcanvasRealTwo[view].delete("tag")
 self.AllcanvasvirtTwo[view].delete("tag")
 if state == 0:
 self.draw(self.AllcoordinatesReal[view], self.AllcanvasRealTwo[view])
 self.draw(self.AllcoordinatesVirt[view], self.AllcanvasvirtTwo[view])

 def hideDonutsOrPins(self, view):
 *"""
 The function `hideDonutsOrPins` takes in a view and hides either donuts or pins based on the user's selection.

 """* hideDonuts = self.AllhideDonuts_btn[view].get()
 hidePins = self.AllhidePins_btn[view].get()
 canvasRealTwo = self.AllcanvasRealTwo[view]
 canvasRealTwoDonutsOrPins = self.AllcanvasRealTwoDonutsOrPins[view]
 canvasvirtTwo = self.AllcanvasvirtTwo[view]
 canvasvirttwoDonutsOrPins = self.AllcanvasvirtTwoDonutsOrPins[view]

 img = self.AllImg[view]
 drr = self.AllCurrentDRR[view]
 RegisterdPins = self.AllRegisterdPins[view]
 RegisterdDonuts = self.AllRegisterdDonuts[view]
 RegisterdTogether = self.AllRegisterdTogether[view]
 Pins = self.AllPins[view]
 Donuts = self.AllDonuts[view]
 Together = self.AllTogether[view]
 if hideDonuts and hidePins:
 canvasRealTwo.itemconfig(canvasRealTwoDonutsOrPins, image=img)
 canvasRealTwo.image = img
 canvasvirtTwo.itemconfig(canvasvirttwoDonutsOrPins, image=drr)
 canvasvirtTwo.image = drr

 if hideDonuts and not hidePins:
 canvasRealTwo.itemconfig(canvasRealTwoDonutsOrPins, image=RegisterdPins)
 canvasRealTwo.image = RegisterdPins
 canvasvirtTwo.itemconfig(canvasvirttwoDonutsOrPins, image=Pins)
 canvasvirtTwo.image = Pins

 if hidePins and not hideDonuts:
 canvasRealTwo.itemconfig(canvasRealTwoDonutsOrPins, image=RegisterdDonuts)
 canvasRealTwo.image = RegisterdDonuts
 canvasvirtTwo.itemconfig(canvasvirttwoDonutsOrPins, image=Donuts)
 canvasvirtTwo.image = Donuts

 if not hidePins and not hideDonuts:
 canvasRealTwo.itemconfig(canvasRealTwoDonutsOrPins, image=RegisterdTogether)
 canvasRealTwo.image = RegisterdTogether
 canvasvirtTwo.itemconfig(canvasvirttwoDonutsOrPins, image=Together)
 canvasvirtTwo.image = Together

 def registerpoints(self, view):
 *"""
 The function `registerpoints` performs image registration and creates visualizations of the registered images.
 1. A point to point registration is performed
 2. The images are converted to a transparent image in either blue or red, or combined
 3. An affine warp of the image is performed based on the point to point registration
 4. The images are saved and shown to the user
 5. The error is calculated and shown to the user


 """* virtNP = np.array(self.AllcoordinatesVirt[view])
 realNP = np.array(self.AllcoordinatesReal[view])
 d, Z, tform, T, b, c = procrustesfunc(realNP, virtNP, scaling=True, reflection='best')
 for x in range(len(self.AllcoordinatesVirt[view])):
 self.AllcanvasRealTwo[view].create_text(Z[x, 0], (Z[x, 1] - 4), fill='blue', font=('Helvetica 15 bold'), text="> " + str(x + 1), tag="tag")
 img1 = self.load_image(self.patientScriptingDir + str("/" + self.AllImageNames[view] + "_" + self.DRRNamesSingle[0]) + '.bmp', photo_img=False, crop=self.Allcrop[view], rotate=self.rotate[view], flip=self.flipimg[view])
 img2 = self.load_image(self.patientScriptingDir + str("/" + self.AllImageNames[view] + "_" + self.DRRNamesSingle[1]) + '.bmp', photo_img=False, crop=self.Allcrop[view], rotate=self.rotate[view], flip=self.flipimg[view])
 img1 = img1.convert("RGBA")
 datas = img1.getdata()
 newData = []
 for item in datas:
 if item[0] == 255 and item[1] == 255 and item[2] == 255:
 newData.append((0, 0, 0, 0)) *# This is for checking white pixels, replace transparent. Do
 # if item[3] == 0 to check for transparent pixels.* else:
 newData.append((255 - item[0], 0, 0, 255 - item[0]))
 img1.putdata(newData)
 img2 = img2.convert("RGBA")
 datas = img2.getdata()
 newData = []
 for item in datas:
 if item[0] == 255 and item[1] == 255 and item[2] == 255:
 newData.append((0, 0, 0, 0)) *# This is for checking white pixels, replace transparent. Do
 # if item[3] == 0 to check for transparent pixels.* else:
 newData.append((0, 0, 255 - item[0], 255 - item[0]))
 img2.putdata(newData)
 img3 = Image.alpha_composite(img1, img2)
 self.AllPins[view] = ImageTk.PhotoImage(img1)
 self.AllDonuts[view] = ImageTk.PhotoImage(img2)
 self.AllTogether[view] = ImageTk.PhotoImage(img3)
 image_array1 = np.array(img1)
 image_array2 = np.array(img2)
 image_array3 = np.array(img3)

 matrix = np.zeros(shape=(2, 3))
 matrix2 = np.dot(b, T)
 matrix3 = matrix2.transpose((1, 0))
 matrix[:, 0] = matrix3[:, 0]
 matrix[:, 1] = matrix3[:, 1]
 matrix[:, 2] = c[:]
 *#* translated_image1 = cv2.warpAffine(src=image_array1, M=matrix, dsize=(self.imageSize, self.imageSize), borderMode=cv2.BORDER_TRANSPARENT)
 translated_image1 = Image.fromarray(translated_image1)
 translated_image2 = cv2.warpAffine(src=image_array2, M=matrix, dsize=(self.imageSize, self.imageSize), borderMode=cv2.BORDER_TRANSPARENT)
 translated_image2 = Image.fromarray(translated_image2)
 translated_image3 = cv2.warpAffine(src=image_array3, M=matrix, dsize=(self.imageSize, self.imageSize), borderMode=cv2.BORDER_TRANSPARENT)
 translated_image3 = Image.fromarray(translated_image3)
 *#* intraopimg = self.AllImgNonphoto[view].convert("RGBA")
 img1 = Image.alpha_composite(intraopimg, translated_image1.convert("RGBA"))
 img2 = Image.alpha_composite(intraopimg, translated_image2.convert("RGBA"))
 img1.save(self.patientScriptingDir + r'/intraopimage' + str(view) + '_1.png', format='png')
 img2.save(self.patientScriptingDir + r'/intraopimage' + str(view) + '_2.png', format='png')

 self.AllRegisterdPins[view] = ImageTk.PhotoImage(translated_image1)
 self.AllRegisterdDonuts[view] = ImageTk.PhotoImage(translated_image2)
 self.AllRegisterdTogether[view] = ImageTk.PhotoImage(translated_image3)
 self.AllcanvasRealTwoDonutsOrPins[view] = self.AllcanvasRealTwo[view].create_image(self.imageSize / 2, self.imageSize / 2, image=self.AllImg[view])
 self.AllcanvasRealTwo[view].itemconfig(self.AllcanvasRealTwoDonutsOrPins[view], image=self.AllRegisterdTogether[view])
 self.AllcanvasRealTwo[view].image = self.AllRegisterdTogether[view]

 self.AllcanvasvirtTwoDonutsOrPins[view] = self.AllcanvasvirtTwo[view].create_image(self.imageSize / 2, self.imageSize / 2, image=self.AllCurrentDRR[view])
 self.AllcanvasvirtTwo[view].itemconfig(self.AllcanvasvirtTwoDonutsOrPins[view], image=self.AllTogether[view])
 self.AllcanvasvirtTwo[view].image = self.AllTogether[view]

 euclideanDistance = []
 for x in range(len(realNP[:, 0])):
 dist = np.linalg.norm(realNP[x, :] - Z[x, :])
 euclideanDistance.append(dist)

 maxDis = np.amax(euclideanDistance)
 meanDis = np.mean(euclideanDistance)
 outputlabel = customtkinter.CTkLabel(self.AllRegistrationframes[view], text="Normalized error: " + '{:0.3f}'.format(d * 100))
 outputlabelmax = customtkinter.CTkLabel(self.AllRegistrationframes[view], text="Max distance: " + '{:0.2f}'.format(maxDis) + " pixels, " + '{:0.2f}'.format(maxDis * 0.3) + " mm")
 outputlabelmean = customtkinter.CTkLabel(self.AllRegistrationframes[view], text="Mean distance: " + '{:0.2f}'.format(meanDis) + " pixels, " + '{:0.2f}'.format(meanDis * 0.3) + " mm")

 outputlabel.grid(row=1, column=4, sticky=tkinter.W)
 outputlabelmax.grid(row=2, column=4, sticky=tkinter.W)
 outputlabelmean.grid(row=3, column=4, sticky=tkinter.W)

 def export_view(self, f, visualised_objects, name, imageSize=800):
 f.simulate(objects_contrast=visualised_objects)
 filename = self.patientScriptingDir + '/' + name + ".bmp"
 *# objs = [p for p in mimics.data.points]
 #
 # mimics.view.export_simulated_fluoroscopy(filename, f, colored_objects=objs, width=imageSize, height=imageSize,
 # image_type='autodetect', )* print(f"{filename} exported")

 def make_powerpoint(self):
 *"""
 The function `make_powerpoint` creates a PowerPoint presentation and adds multiple slides with images to it.
 """* left, top = Cm(14.82), Cm(0)
 filename = self.patientScriptingDir + r'/3DP-' + str(self.driedpnummer) + '_OK_Slides_iFuse_' + str(self.side) + '_' + str(
 self.patientNumber) + '.pptx'
 prs = Presentation(filename)
 slide_layout = prs.slide_layouts[1]
 for view in range(3):
 for i in range(2):
 slide = prs.slides.add_slide(slide_layout)
 slide.shapes.add_picture(self.patientScriptingDir + r'/intraopimage' + str(view) + '_' + str(i + 1) + '.png', left, top, width=pptx.util.Inches(7.5), height=pptx.util.Inches(7.5))
 filename = self.patientScriptingDir + r'/3DP-' + str(self.driedpnummer) + '_OK_Slides_iFuse_' + str(self.side) + '_' + str(
 self.patientNumber) + 'Intraopeartieve.pptx'
 prs.save(filename)

 print("saving all images to powerpoint")
 self.wm_state('iconic')

 def minimize(self):
 *"""
 The above function minimizes the window.
 """* self.attributes('-fullscreen', False)
 print("Window is minimized")


*#
#*if __name__ == "__main__":
 app = App()
 app.mainloop()
