# Supplementary material for: Sacroiliac joint fusion guided by intraoperatively superimposed virtual surgical planning using simulated fluoroscopic images
Source: Brain Spine. 2024 Aug 2;4:102905. doi: 10.1016/j.bas.2024.102905 (PMC11386036; doi:10.1016/j.bas.2024.102905)
Supplement: Multimedia component 2 [file mmc2.doc]

*# per operatieve test*import time
import numpy as np
import tkinter as tk
import os


*# create a popup gui to enter the patient reference number*def get_case_nr():
 global caseNumber
 caseNumber = entry1.get()
 root.destroy()


root = tkinter.Tk()
root.title('Fil in the patient reference number')
canvas1 = tkinter.Canvas(root, width=400, height=300)
canvas1.pack()
entry1 = tkinter.Entry(root)
canvas1.create_window(200, 140, window=entry1)
button1 = tkinter.Button(text='OK', command=get_case_nr)
canvas1.create_window(200, 180, window=button1)
root.mainloop()

print(f'Patient rerfernce number is: {caseNumber}')

*# Check of if directory for this patient exist in database*root_dir = os.path.abspath(r'ROOTPATIENTFILE')
root_dir_bestanden = os.path.abspath(r'ROOTHELPSCRIPTS')

patientDir = root_dir + r'\3DP-' + str(caseNumber)
Check = os.path.isdir(patientDir)

if Check == True:
 print(f"path found")
else:
 print(f"Error: path not found")
 raise SystemExit

names = ["Lateraal", "Inlet", "Outlet"]
createdView = [0, 0, 0]
FluoroscopicViews = [[], [], []]
cran_caud = [0, 0, 0]
lao_rao = [0, 0, 0]
AngleArraySize = 5 *# this is the angle in degrees in one direction*AngleArrayStep = 0.5 *# difference in degrees in one step*AngleArraySizeInOutlet = 10 *# this is the angle in degrees in one direction*AngleArrayStepInOutlet = 1 *# difference in degrees in one step*totalImages = (((AngleArraySize * 2) / AngleArrayStep) + 1) ** 2

*# Three masks are created with different HU values to be used to create the virtual fluoroscopic image*pelvis1 = mimics.segment.create_mask()
pelvis1.name = "PelvisHigh"
low_gv = mimics.segment.HU2GV(500)
high_gv = mimics.segment.HU2GV(30000)
mimics.segment.threshold(mask=pelvis1, threshold_min=low_gv, threshold_max=high_gv)

pelvis2 = mimics.segment.create_mask()
pelvis2.name = "PelvisMed"
low_gv = mimics.segment.HU2GV(255)
high_gv = mimics.segment.HU2GV(499)
mimics.segment.threshold(mask=pelvis2, threshold_min=low_gv, threshold_max=high_gv)

pelvis3 = mimics.segment.create_mask()
pelvis3.name = "PelvisLow"
low_gv = mimics.segment.HU2GV(200)
high_gv = mimics.segment.HU2GV(254)
mimics.segment.threshold(mask=pelvis3, threshold_min=low_gv, threshold_max=high_gv)

p = mimics.segment.calculate_part(mask=pelvis1, quality='Optimal')
p.name = pelvis1.name
p.visible = False

p2 = mimics.segment.calculate_part(mask=pelvis2, quality='Optimal')
p2.name = pelvis2.name
p2.visible = False

p3 = mimics.segment.calculate_part(mask=pelvis3, quality='Optimal')
p3.name = pelvis3.name
p3.visible = False


def exportView(f, visualised_objects, name, imageSize=800):
 *"""
 The function ExportView takes in a fluoroscopic view and exports the image at a prefered size

 :param f: This is the fluoroscopic view
 :param visualised_objects: All the objects that need to ve visualized on the image
 :param name: save name of the image
 """* f.simulate(objects_contrast=visualised_objects)
 filename = new_dir + '/' + name + ".bmp"
 objs = [p for p in mimics.data.points]

 mimics.view.export_simulated_fluoroscopy(filename, f, colored_objects=objs, width=imageSize, height=imageSize,
 image_type='autodetect', )
 print(f"{filename} exported")


*# The previously created fluoroscopic images are searched en saved in the variable*for i in range(3):
 f = mimics.data.fluoroscopy_views.find(names[i])
 FluoroscopicViews[i] = f

viewNumber = 0
FluoroscopicViews[viewNumber].attenuation_coefficient = 0

*# create different object list to be used to visualise them in the virtual image*visualised_objectsPelvisOnly = []
visualised_objectsGuidepinsOnly = []
visualised_objectsDonutsOnly = []
for p in mimics.data.parts:
 if p.name == "Pelvis":
 visualised_objectsPelvisOnly.append((p, 0.06))
 elif p.name == "Implantaten_insitu":
 visualised_objectsPelvisOnly.append((p, 0.6))
 elif p.name == "PelvisHigh":
 visualised_objectsPelvisOnly.append((p, 0.1))
 elif p.name == "PelvisMed" or p.name == "PelvisLow":
 visualised_objectsPelvisOnly.append((p, 0.06))
 elif p.name == "Kdraad Craniaal" or p.name == "Kdraad Middel" or p.name == "Kdraad Caudaal":
 visualised_objectsGuidepinsOnly.append((p, 0.30))
 elif p.name == "Donut Craniaal" or p.name == "Donut Middel" or p.name == "Donut Caudaal":
 visualised_objectsDonutsOnly.append((p, 2))

DRRNamesSingle = ["Pelvisonly", "Guidepinsonly", "Donutsonly"]
allObjectes = [visualised_objectsPelvisOnly, visualised_objectsGuidepinsOnly, visualised_objectsDonutsOnly]

*################## Create lateral images #################*LateralAngleArrray = [FluoroscopicViews[viewNumber].lao_rao_angle,
 FluoroscopicViews[viewNumber].cran_caud_angle] *# get the True lateral view as defined in the VSP*cran_caud[viewNumber] = FluoroscopicViews[viewNumber].cran_caud_angle
FluoroscopicViews[viewNumber].cran_caud_angle = cran_caud[
 viewNumber] - AngleArraySize *# set the view to the starting position*lao_rao[viewNumber] = FluoroscopicViews[viewNumber].lao_rao_angle
FluoroscopicViews[viewNumber].lao_rao_angle = lao_rao[
 viewNumber] - AngleArraySize *# set the view to the starting position

# create a new dir to save all the lateral images*path = os.path.join(new_dir, r'LatImageArray')
Check = os.path.isdir(path)
if Check == False:
 mode = 0o666
 os.mkdir(path, mode)
old_dir = new_dir *# with scripting*new_dir = path *# inside imageArray directory*N = 1
start = time.time()

*# iteratively create an image, change the angle and repeat*for i in range(int(((AngleArraySize * 2) / AngleArrayStep) + 1)):
 for j in range(int(((AngleArraySize * 2) / AngleArrayStep) + 1)):
 exportView(FluoroscopicViews[viewNumber], visualised_objectsPelvisOnly, str(N), imageSize=800)
 FluoroscopicViews[viewNumber].lao_rao_angle = FluoroscopicViews[viewNumber].lao_rao_angle + AngleArrayStep
 N += 1
 FluoroscopicViews[viewNumber].lao_rao_angle = FluoroscopicViews[viewNumber].lao_rao_angle - (
 AngleArraySize * 2) - AngleArrayStep
 FluoroscopicViews[viewNumber].cran_caud_angle = FluoroscopicViews[viewNumber].cran_caud_angle + AngleArrayStep
 print(f"Current image is {N}/{int(totalImages)}, time elapsed is {int((time.time() - start) / 60)} min")

new_dir = old_dir *# revert back to scripting directory

# Set the views to the True lateral views as defined in the VSP*FluoroscopicViews[viewNumber].lao_rao_angle = LateralAngleArrray[0]
FluoroscopicViews[viewNumber].cran_caud_angle = LateralAngleArrray[1]

*################## Create inlet images #################*viewNumber = 1
InletAngleArrray = [FluoroscopicViews[viewNumber].lao_rao_angle, FluoroscopicViews[viewNumber].cran_caud_angle]
cran_caud[viewNumber] = FluoroscopicViews[viewNumber].cran_caud_angle
FluoroscopicViews[viewNumber].cran_caud_angle = cran_caud[viewNumber] - AngleArraySizeInOutlet

path = os.path.join(new_dir, r'InletImageArray')
Check = os.path.isdir(path)
if Check == False:
 mode = 0o666
 os.mkdir(path, mode)
old_dir = new_dir *# with scripting*new_dir = path *# inside imageArray directory*N = 1
start = time.time()

for j in range(int(((AngleArraySizeInOutlet * 2) / AngleArrayStepInOutlet) + 1)):
 exportView(FluoroscopicViews[viewNumber], visualised_objectsPelvisOnly, str(N), imageSize=800)
 FluoroscopicViews[viewNumber].cran_caud_angle = FluoroscopicViews[
 viewNumber].cran_caud_angle + AngleArrayStepInOutlet
 N += 1
FluoroscopicViews[viewNumber].cran_caud_angle = FluoroscopicViews[viewNumber].cran_caud_angle - (AngleArraySizeInOutlet)
print(f"All Inlet images are created, time elapsed is {int((time.time() - start) / 60)} min")

new_dir = old_dir *# revert back to scripting directory*FluoroscopicViews[viewNumber].lao_rao_angle = InletAngleArrray[0]
FluoroscopicViews[viewNumber].cran_caud_angle = InletAngleArrray[1]

*################## Create outlet images #################*viewNumber = 2
OutletAngleArrray = [FluoroscopicViews[viewNumber].lao_rao_angle, FluoroscopicViews[viewNumber].cran_caud_angle]
cran_caud[viewNumber] = FluoroscopicViews[viewNumber].cran_caud_angle
FluoroscopicViews[viewNumber].cran_caud_angle = cran_caud[viewNumber] - AngleArraySizeInOutlet

path = os.path.join(new_dir, r'OutletImageArray')
Check = os.path.isdir(path)
if Check == False:
 mode = 0o666
 os.mkdir(path, mode)
old_dir = new_dir *# with scripting*new_dir = path *# inside imageArray directory*N = 1
start = time.time()

for j in range(int(((AngleArraySizeInOutlet * 2) / AngleArrayStepInOutlet) + 1)):
 exportView(FluoroscopicViews[viewNumber], visualised_objectsPelvisOnly, str(N), imageSize=800)
 FluoroscopicViews[viewNumber].cran_caud_angle = FluoroscopicViews[
 viewNumber].cran_caud_angle + AngleArrayStepInOutlet
 N += 1
FluoroscopicViews[viewNumber].cran_caud_angle = FluoroscopicViews[viewNumber].cran_caud_angle - (AngleArraySizeInOutlet)
print(f"All Outlet images are created, time elapsed is {int((time.time() - start) / 60)} min")

new_dir = old_dir *# revert back to scripting directory*FluoroscopicViews[viewNumber].lao_rao_angle = OutletAngleArrray[0]
FluoroscopicViews[viewNumber].cran_caud_angle = OutletAngleArrray[1]

viewAngles = [LateralAngleArrray, InletAngleArrray, OutletAngleArrray]
np.savetxt(new_dir + r'\Trueangles.txt', viewAngles, delimiter=',')
